# Supplementary material for: Metabolite interactions in the bacterial Calvin cycle and implications for flux regulation
Source: Commun Biol. 2023 Sep 18;6:947. doi: 10.1038/s42003-023-05318-8 (PMC10507043; doi:10.1038/s42003-023-05318-8)
Supplement: Supplementary file 2 — Supplementary Material [file 42003_2023_5318_MOESM2_ESM.pdf]

Supplementary Material

Metabolite interactions in the bacterial Calvin cycle and implications for flux regulation
Sporre *et al.*

List of Supplementary Figures and Tables

Fig. S1. Change in LiP digestion upon reduction and oxidation of *Synechocystis* proteome
Fig. S2: Number of detected peptides in every LiP-SMap experiment
Fig. S3: Number of peptides detected per metabolite-interacting protein compared to non-interacting proteins
Fig. S4: Overlap and correlation of peptides from repeat LiP-SMap experiments
Fig. S5: Persistence of interactions at low metabolite concentrations at high metabolite concentrations
Fig. S6: Fraction interacting orthologs within functional groups in each organism
Fig. S7: Similarity of ortholog interaction patterns (low concentration)
Fig. S8: Log2(fold change) and significance of detected proteins in presence of 2 mM ATP at different Mg^{2+} concentrations
Fig. S9: Interactions of Calvin cycle enzymes and selected central carbon metabolism enzymes with metabolites (low concentration, $q<0.05$)
Fig. S10: Effect of DHAP and GAP on the catalytic activity of syn-F/SBPase
Fig. S11: Glyceraldehyde-3-phosphate (GAP) effect on thermal stability of *Synechocystis* F/SBPase at different Mg^{2+} concentrations
Fig. S12: Thermostability assays of *Cupriavidus* F/SBPase in the presence of various metabolites
Fig. S13: Thermostability assays of *Synechocystis* F/SBPase in the presence of various metabolites
Fig. S14: End-point *in vitro* assay of cnF/SBPase in presence of G6P
Fig. S15: LiP-Smap of purified syn-F/SBPase treated with GAP and NADPH
Fig. S16: Light scattering assays of syn-F/SBPase under various conditions
Fig. S17: Measured concentrations of product after 20 minutes of *in vitro* reaction as detected by malachite green assay and LC/MS
Fig. S18: Effect of different metabolites (1 mM) on the kinetics of *Cupriavidus* transketolase
Fig. S19: Effect of different metabolites (1 mM) on the kinetics of *Synechocystis* transketolase
Fig. S20: Thermostability assays of *Synechocystis* and *Cupriavidus* transketolase in the presence of various metabolites
Fig. S21: Kinetic analysis of the *Synechocystis* F/SBPase R194H mutant
Fig. S22: Flux control coefficients for all reactions in the model
Fig. S23: Difference between median FCCs between model variants

Table S1: Chosen concentrations for every used metabolite and their most extreme values found in literature
Table S2: All metabolite concentrations found across 7 metabolomics studies
Table S3: Changes in fructose/sedoheptulose bisphosphatase kinetic parameters in the presence of metabolites

Table S4: Changes in transketolase kinetic parameters in the presence of various metabolites at 1 mM
Table S5: Transition list for mass spectrometry

Supplementary Dataset S1 (separate data file) List of significantly affected proteins in *Synechocystis* PCC 6803 by reduction/oxidation through DTT/DTNB
Supplementary Dataset S2 (separate data file) Table of all detected peptides across LiP-SMap experiments with fold-change and significance statistics; *Synechocystis* PCC 6803
Supplementary Dataset S3 (separate data file) Table of all detected peptides across LiP-SMap experiments with fold-change and significance statistics; *Synechoccocus* PCC 7942
Supplementary Dataset S4 (separate data file) Table of all detected peptides across LiP-SMap experiments with fold-change and significance statistics; *Cupriavidus necator*
Supplementary Dataset S5 (separate data file) Table of all detected peptides across LiP-SMap experiments with fold-change and significance statistics; *Hydrogenophaga pseudoflava*
Supplementary Dataset S6: (separate data file) List of all proteins affected by at least one metabolite and their KEGG orthology groups (KOGs)
Supplementary Dataset S7: (separate data file) List of all significant peptides in LiP-SMap experiment with magnesium and ATP
Supplementary Dataset S8: (separate data file) Phylogenetic trees of Calvin cycle enzymes labeled with detected protein-metabolite interactions
Supplementary Dataset S9: (separate data file) Raw data for kinetic-, melting temperature- and light scattering assays.

Supplementary Datasets S1-S9 are available on figshare.com with DOI 10.6084/m9.figshare.23939604

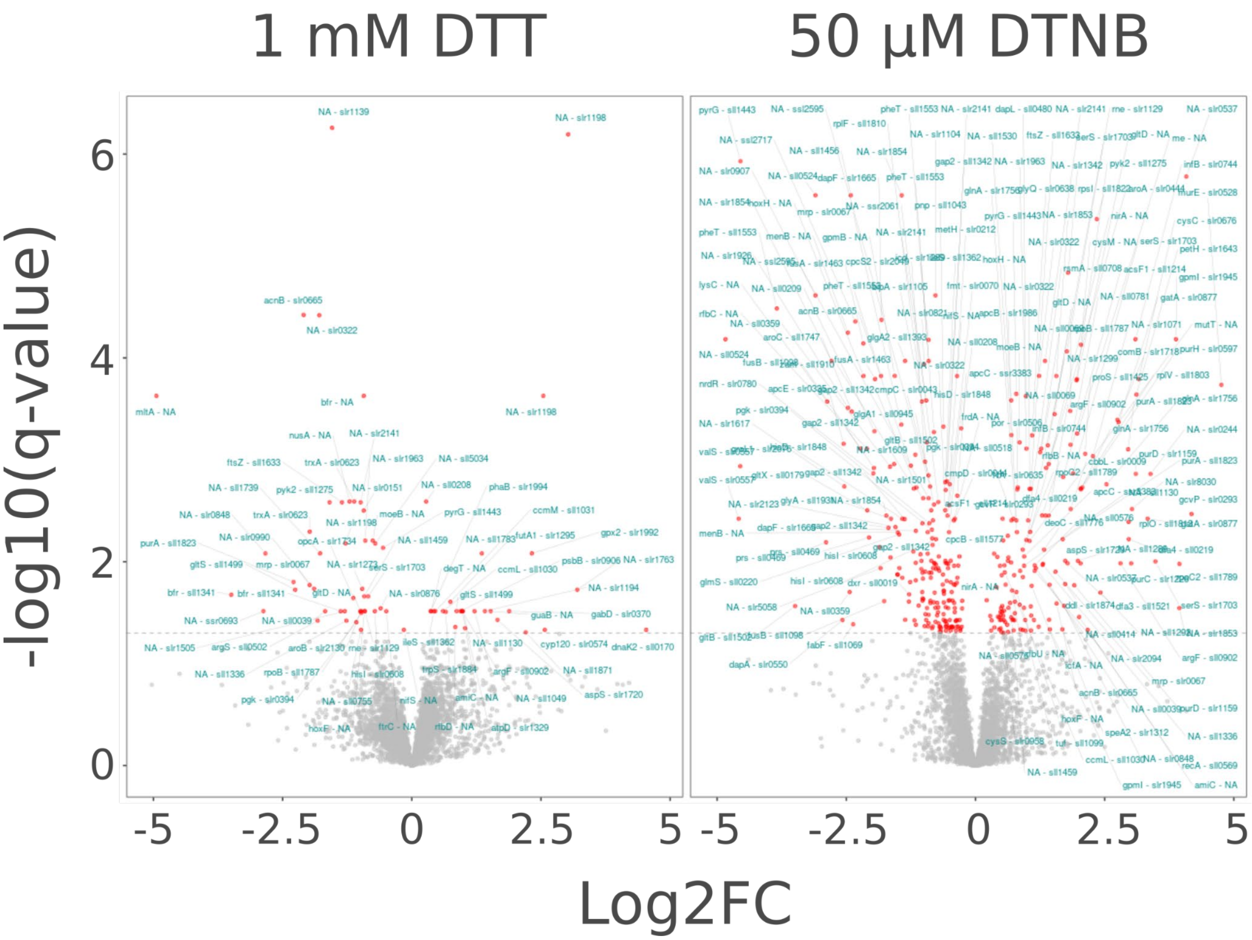

Fig. S1. Change in LiP digestion upon reduction and oxidation of *Synechocystis* proteome. Synechocystis proteome extracts treated with either DTT (reductant) or DTNB (oxidant) were subjected to LiP-SMap analysis (untreated proteome as control). Each point represents a peptide that was detected in both the treated and untreated condition. A point's coordinate on the x-axis corresponds to the peptide abundance fold change (FC), treated vs. untreated. Y-axes show the statistical significance of the fold change. Red points indicate significantly changed proteins ($q < 0.05$), and insets display the number of significantly changed proteins. Full list of peptides, log2FC, and statistical tests in **Supplementary Dataset S1.**

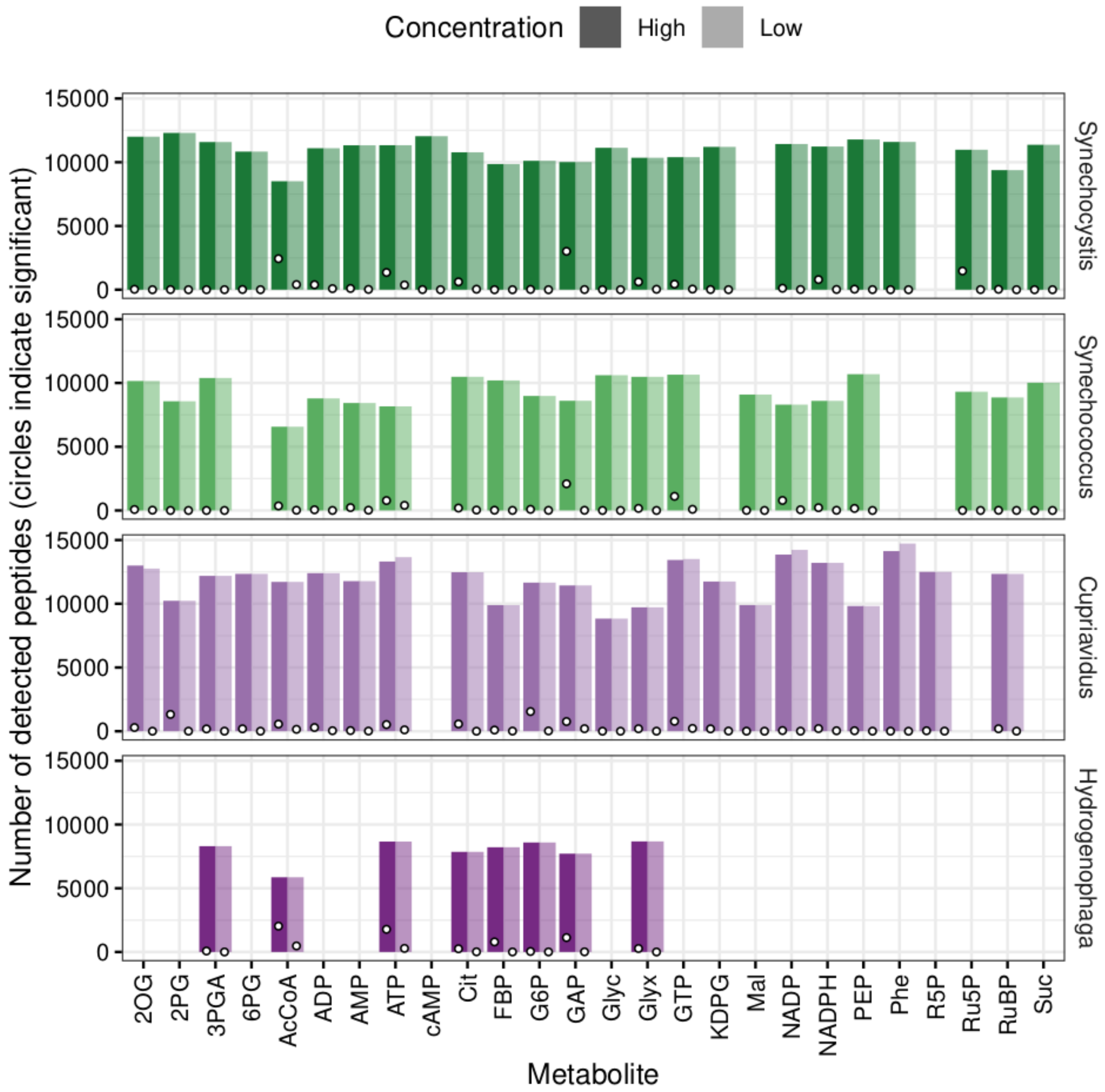

Fig. S2. Number of detected peptides in every LiP-SMap experiment. The darker hue represents the high concentration of added metabolite and the lighter hue represents the low concentration. Circles indicate the number of significant peptides. When a metabolite was not tested against an organism, the space is blank.

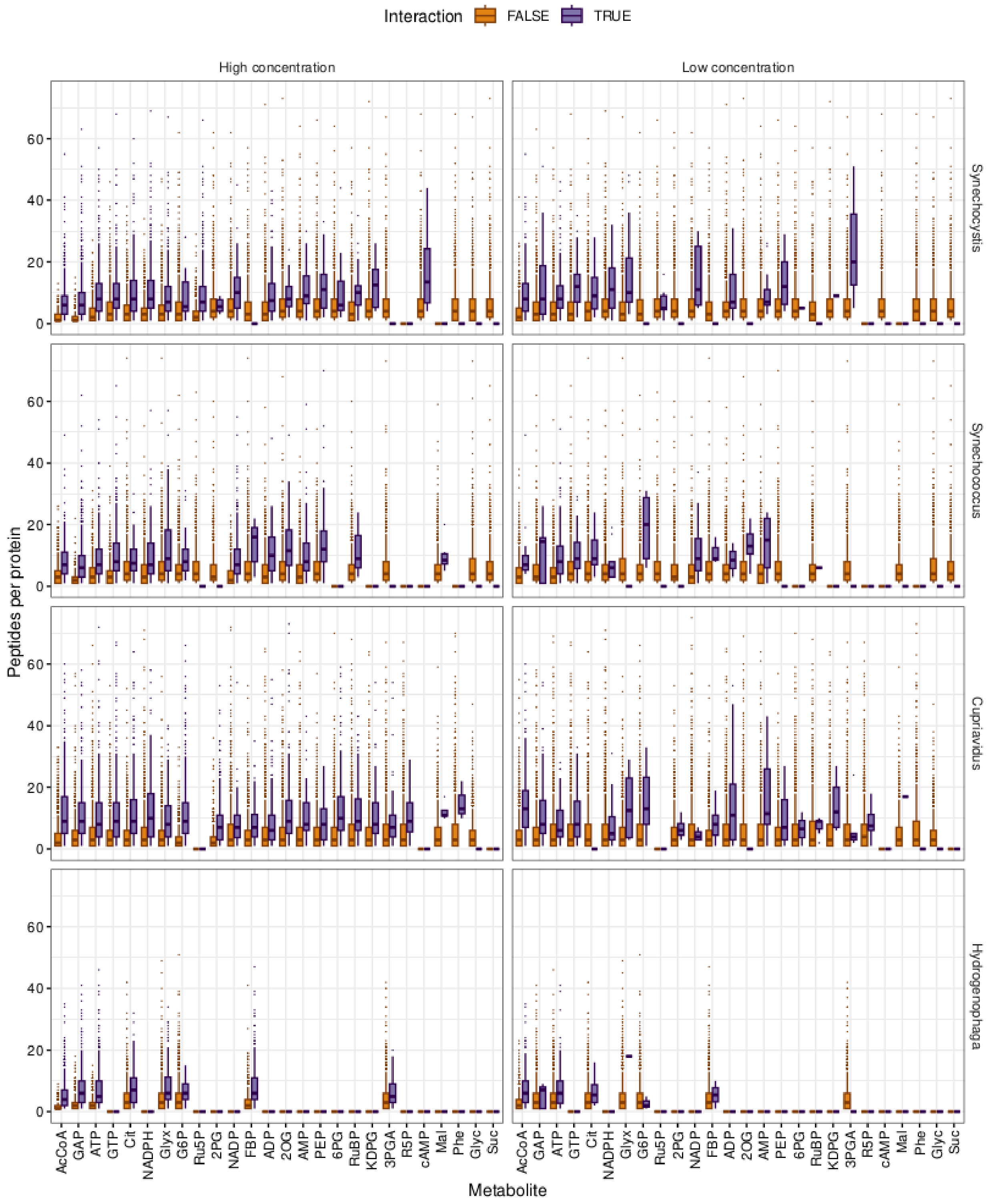

Fig. S3. Number of peptides detected per metabolite-interacting protein compared to non-interacting proteins. Proteins were classified as not having interaction or having interaction with the tested metabolites, *i.e.* “Interaction FALSE” and “Interaction TRUE”. Interaction means that at least one peptide was significantly changed in abundance in presence of the metabolite ($q < 0.01$). The y-axis indicates the number of peptides detected per protein

summarized as box plots. The plots are split by high and low concentration of the interacting metabolite (columns) and by organism (rows).

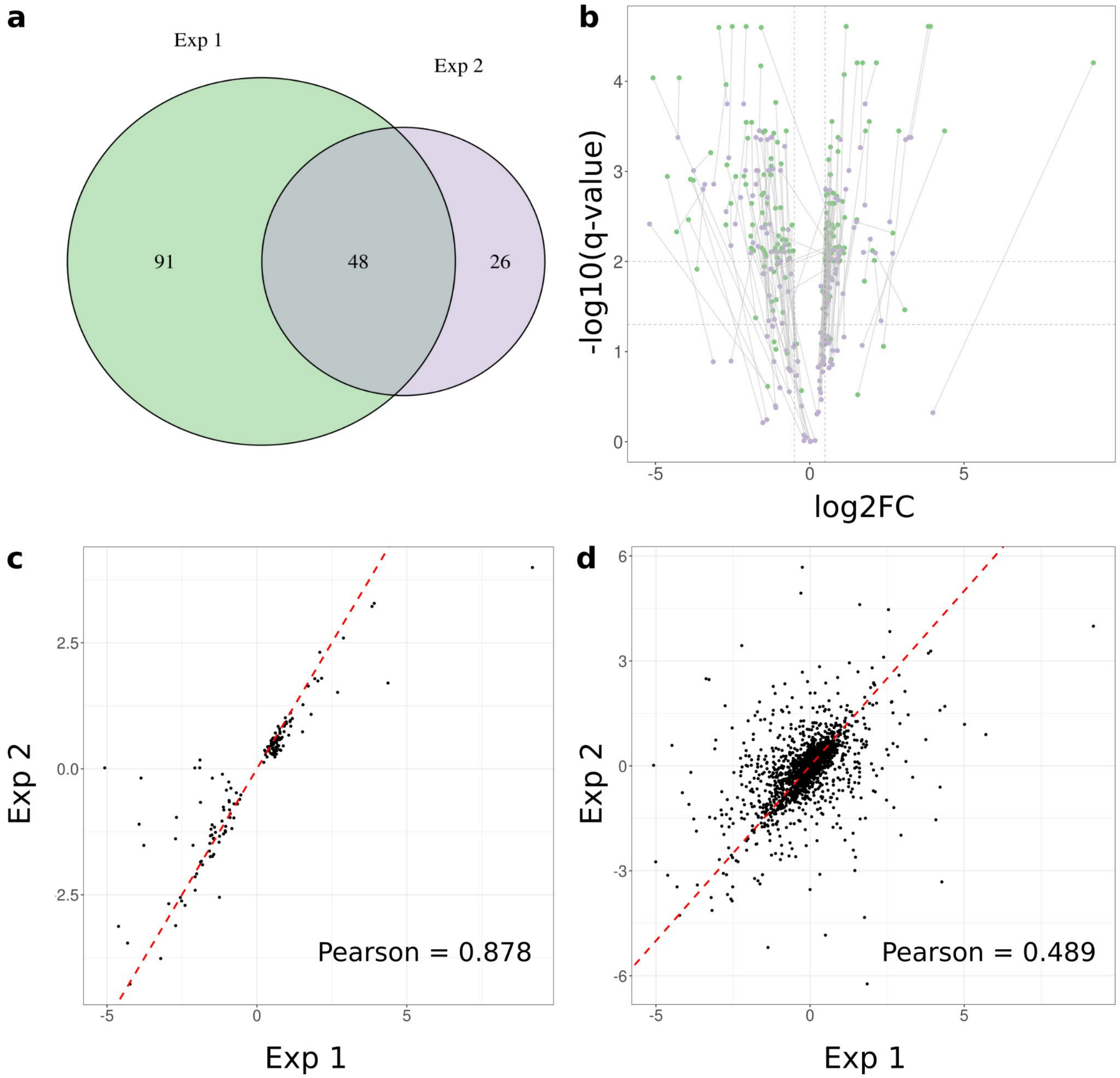

Fig. S4. Overlap and correlation of peptides from repeat *Synechococcus* PCC 7942 LiP-SMap experiments with 10 mM glyoxylate. Two LiP-SMap experiments were performed in parallel, with the same reagent stocks and were run together on the LC-MS. **a** Overlap of significant peptides between the two experiments. **b** Volcano plot of all peptides significant in at least one experiment, with each peptide pair connected by a line. The lines being generally vertical indicate that the experiments differ mainly in significance rather than effect size. **c** Correlation of the log2 fold changes of peptides with a q-value < 0.01 upon treatment with 10 mM glyoxylate **d** Correlation of the log2 fold changes all peptides (no q-value cutoff) upon treatment with 10 mM glyoxylate.

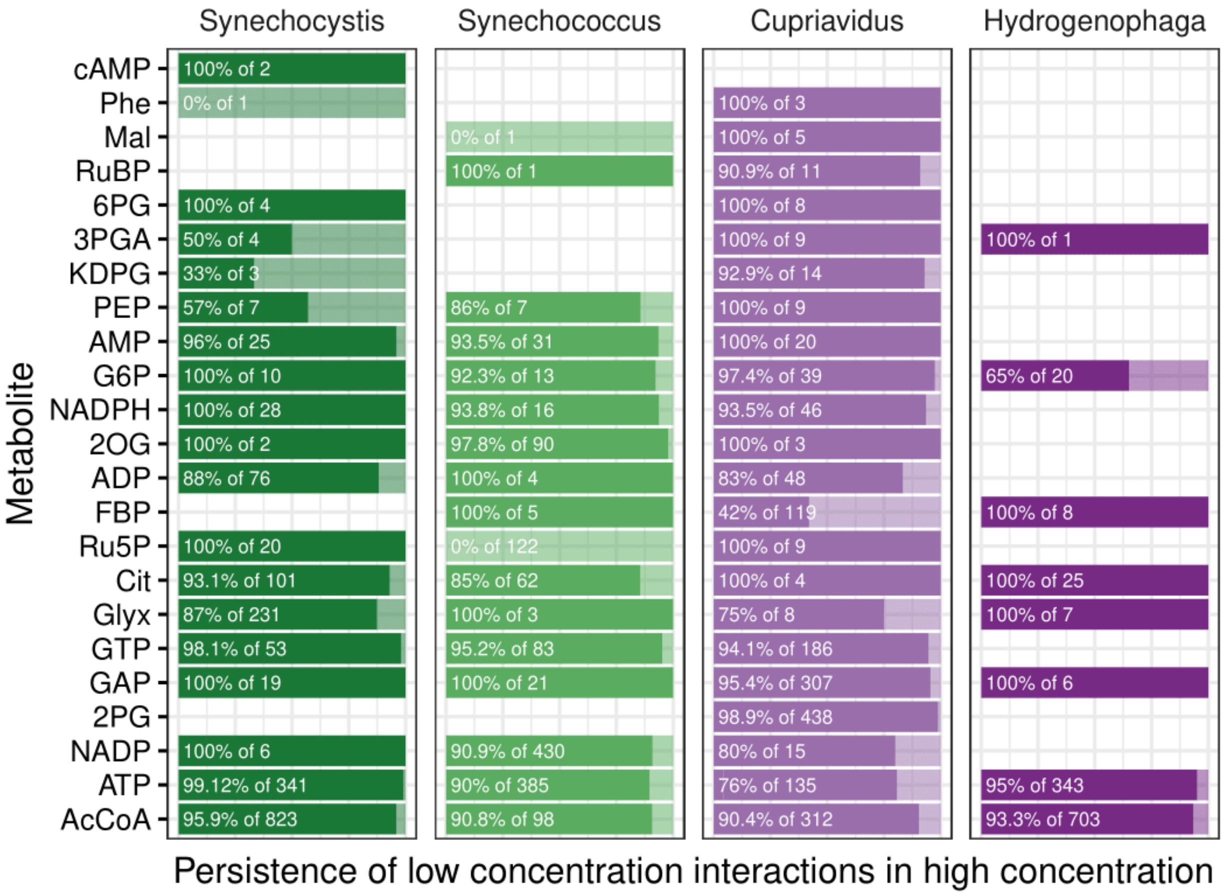

Fig. S5. Persistence of significant interactions at low metabolite concentrations at high metabolite concentrations (q<0.05). Opaque bars indicate the fraction of low concentration interactions that were detected both in the low concentration and high concentration experiments, while transparent bars indicate interactions that were only detected in the low concentration experiments. Metabolites are ordered by the total number of interactions. Metabolites without low concentration interactions are excluded.

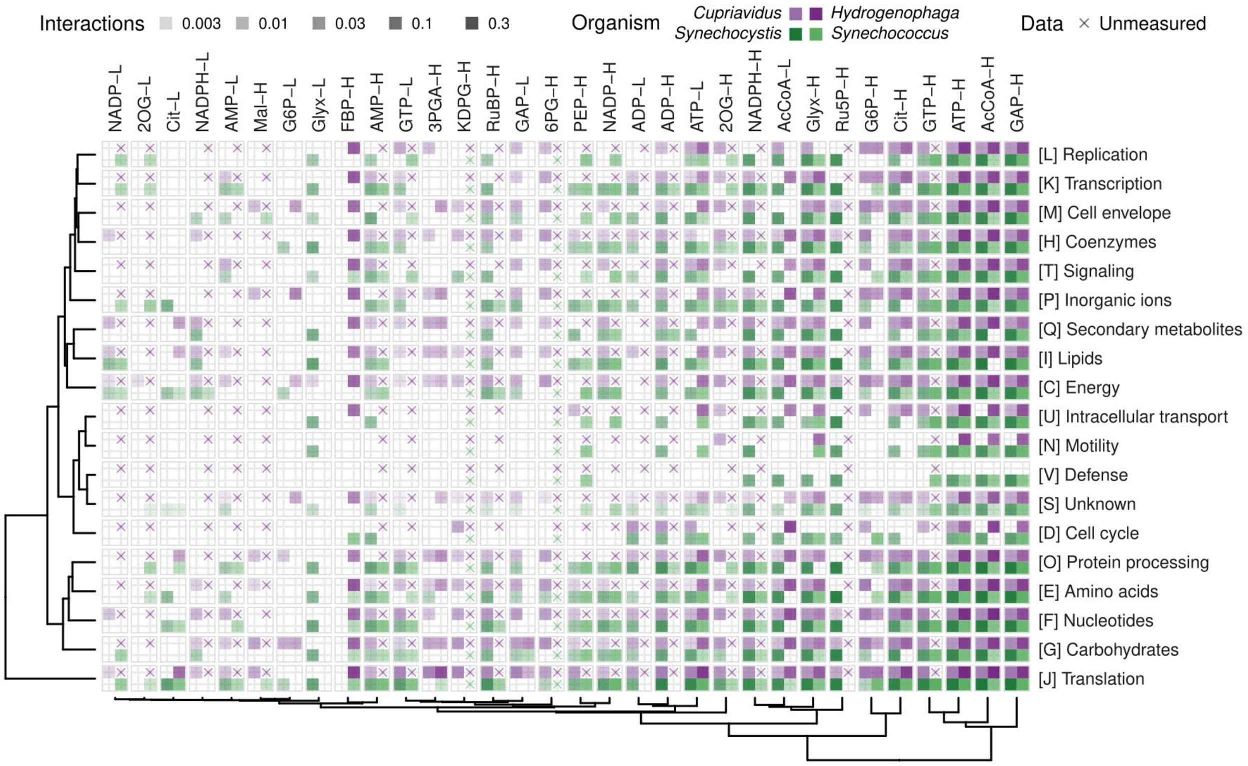

Fig. S6. Fraction interacting orthologs within functional groups in each organism. If at least one sequence per ortholog family interacted with a metabolite at low (L) or high (H) concentration (heatmap columns), that ortholog was considered to be interacting. Interactions were then summarized per functional group (heatmap rows) and normalized by the total number of orthologs in that group. Dendrograms illustrate the clustering patterns of rows and columns based on Euclidean distance and the Ward.D2 algorithm. Interaction fractions in all four organisms contributed both to rows and columns. A cross indicates that the particular condition was not measured.

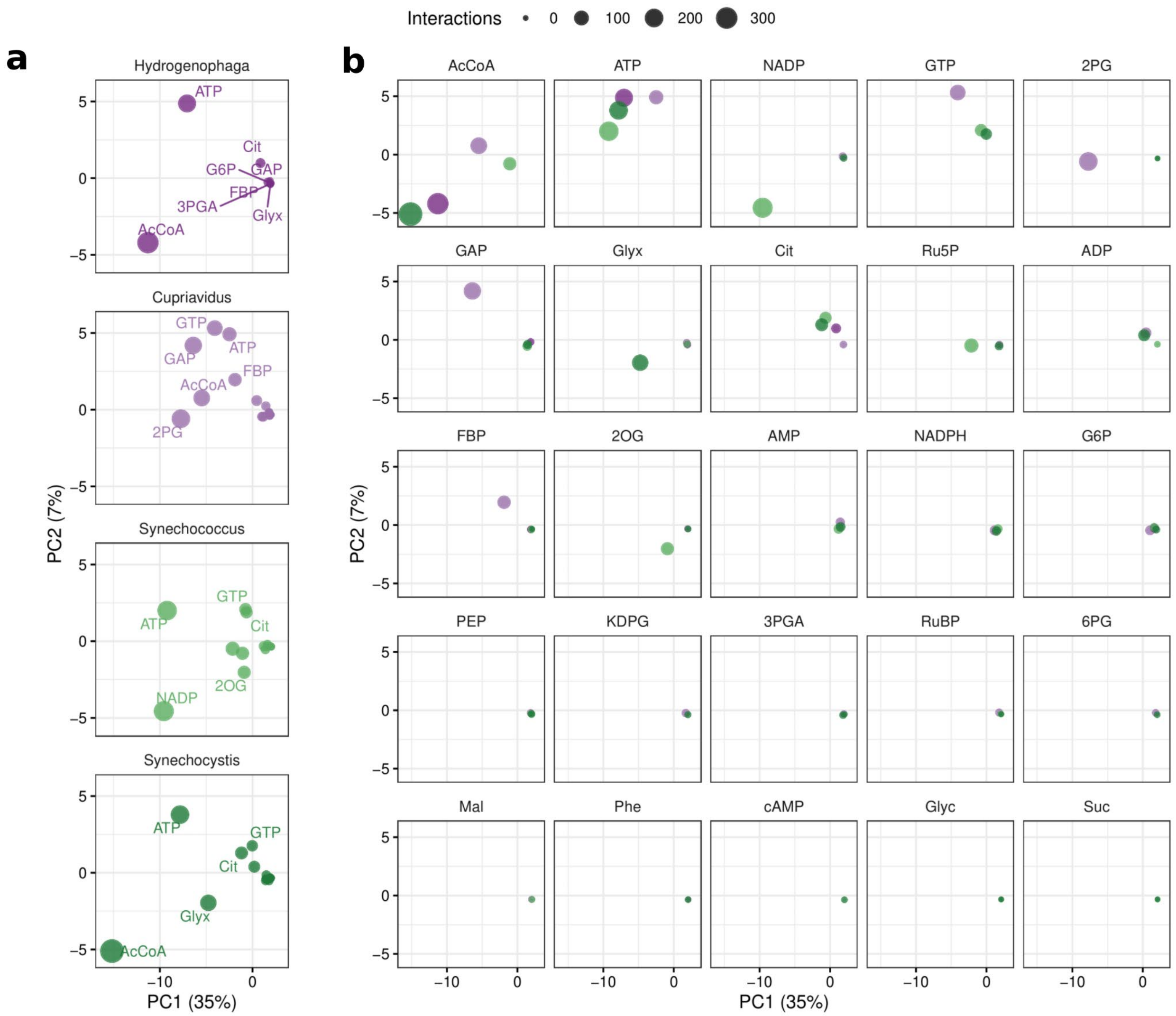

Fig. S7. Similarity of ortholog interaction patterns (low concentration, q<0.05). Principal components were calculated from the presence or absence of interaction with each of 321 orthologs (see Materials and Methods). All data points shown here are from the same principal component analysis, but split per organism **a** or metabolite **b** to reduce overplotting. Percentages indicate the fraction of the total variance captured by the principal components.

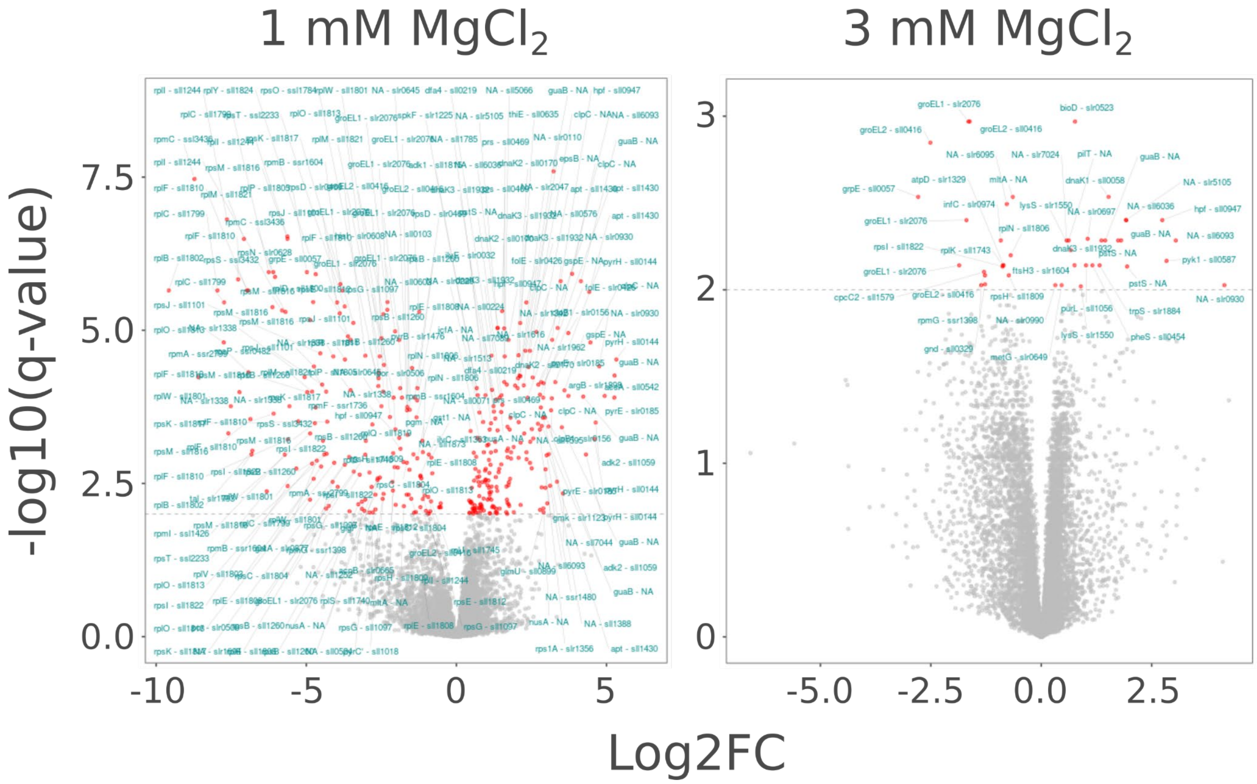

Fig. S8. Log2(fold change) and significance of detected peptides in presence of 2 mM ATP at different Mg^{2+} concentrations. The extracted proteome of *Synechocystis* was treated with 2 mM ATP and either 1 or 3 mM $MgCl_2$ and compared to a sample without ATP but with the same concentration $MgCl_2$. Each peptide detected in both treated and untreated samples are represented by one dot with significantly ($q<0.01$) changed proteins colored in red. A list of all significantly changed peptides in the experiment can be found in **Supplemental Dataset S7.**. The effect of ATP treatment is mitigated by an increased $MgCl_2$ concentration.

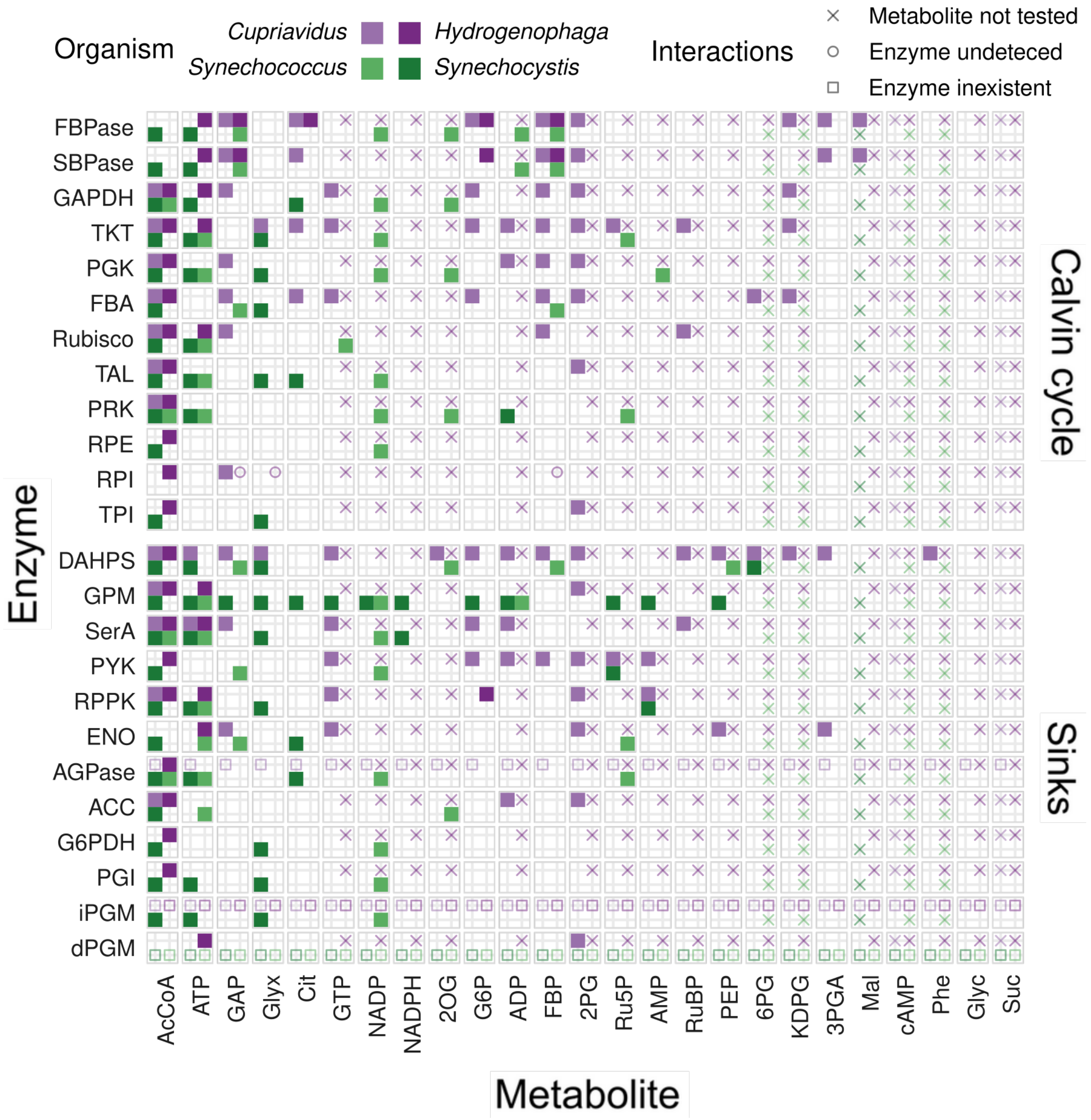

Fig. S9. Interactions of Calvin cycle enzymes and selected central carbon metabolism enzymes with metabolites (low concentration, q<0.05). Interactions between metabolites (columns) at low concentration and enzymes (rows) identified by KEGG EC number annotation are shown for each organism by tiles filled with the corresponding color. A blank tile indicates that the interaction was not detected, while missing protein data is explained by a symbol. A cross indicates that the particular condition was not measured, a circle indicates that no proteins were detected, and a square indicates that there was no such enzyme in the corresponding genome. AGPase, ADP-glucose synthase (EC 2.7.7.27); DAHPS, DAHP synthase (EC 2.5.1.54); dPGM, 2,3-diphosphoglycerate-dependent phosphoglycerate mutase (EC 5.4.2.11); ENO, Enolase (EC 4.2.1.11); FBA, Fructose-bisphosphate aldolase (EC 4.1.2.13); FBPase, Fructose-1,6-bisphosphatase (EC 3.1.3.11); G6PDH, Zwf (EC 1.1.1.49); GAPDH, Glyceraldehyde 3-phosphate dehydrogenase (EC 1.2.1.12, 1.2.1.13, 1.2.1.59); GPM, Phosphoglucomutase (EC 5.4.2.2); iPGM, 2,3-diphosphoglycerate-independent phosphoglycerate

mutase (EC 5.4.2.12); PGI, Phosphoglucoisomerase (EC 5.3.1.9); PGK, Phosphoglycerate kinase (EC 2.7.2.3); PRK, Phosphoribulokinase (EC 2.7.1.19); PYK, Pyruvate kinase (EC 2.7.1.40); RPE, Ribulose-phosphate 3-epimerase (EC 5.1.3.1); RPI, Ribose 5-phosphate isomerase (EC 5.3.1.6); RPPK, Ribose-5-phosphate pyrophosphokinase (EC 2.7.6.1); Rubisco, Ribulose-bisphosphate carboxylase (EC 4.1.1.39); SBPase, Sedoheptulose-1,7-bisphosphatase (EC 3.1.3.37); SerA, Phosphoglycerate dehydrogenase (EC 1.1.1.95); TAL, Transaldolase (EC 2.2.1.2); TKT, Transketolase (EC 2.2.1.1); TPI, Triose-phosphate isomerase (EC 5.3.1.1).

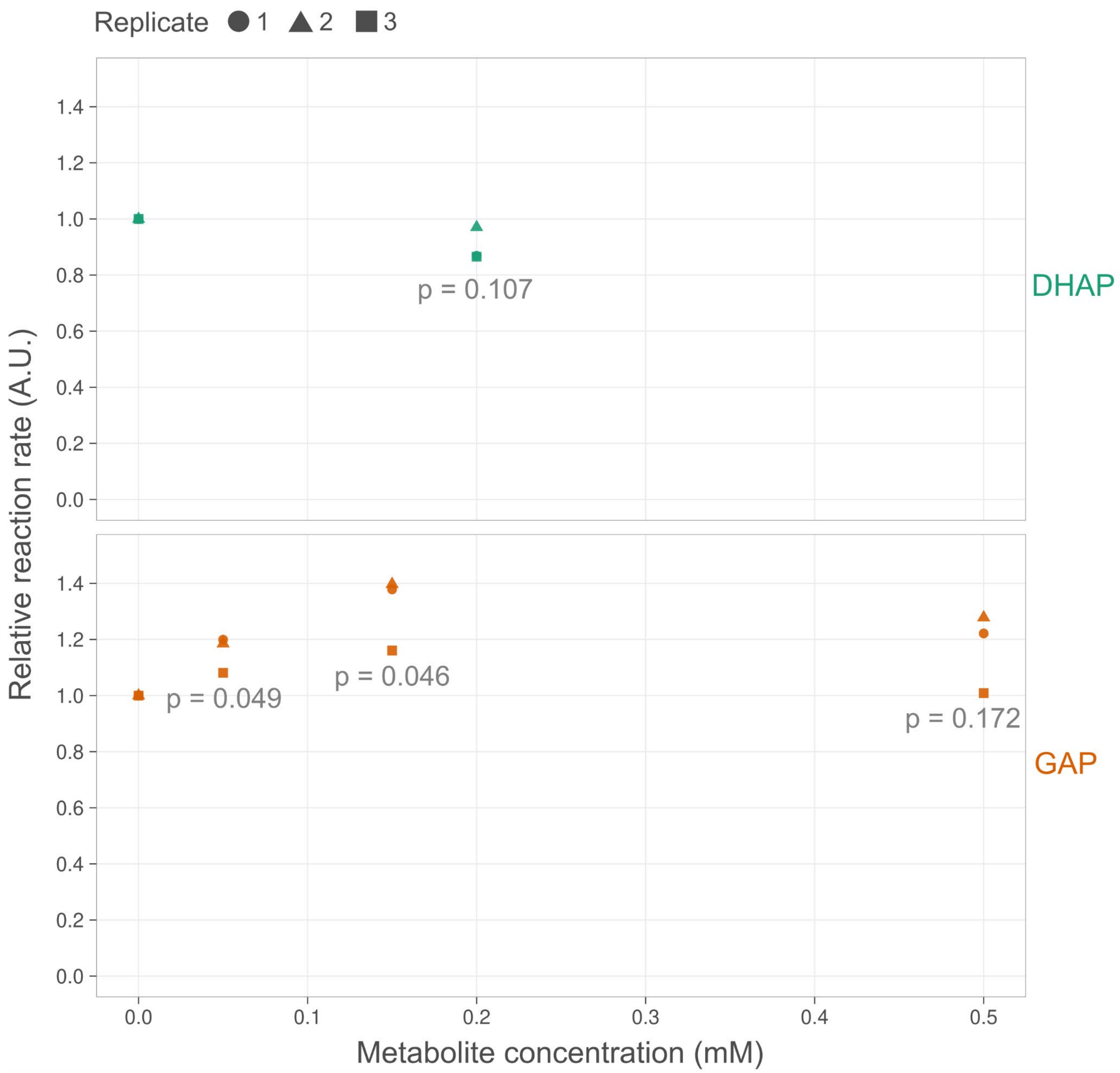

Fig. S10: Effect of DHAP and GAP on the catalytic activity of syn-F/SBPase. The catalytic rate of *Synechocystis* F/SBPase (y-axis) was measured *in vitro* at different DHAP and GAP concentrations (x-axis) under half-saturating substrate concentration (60 µM FBP). Shown initial rates are relative to the rate measured in absence of metabolite e. P-values (p) show the statistical significance of the change in catalytic rate at each tested metabolite concentration relative to the rate at 0 µM metabolite (Student's t-test).

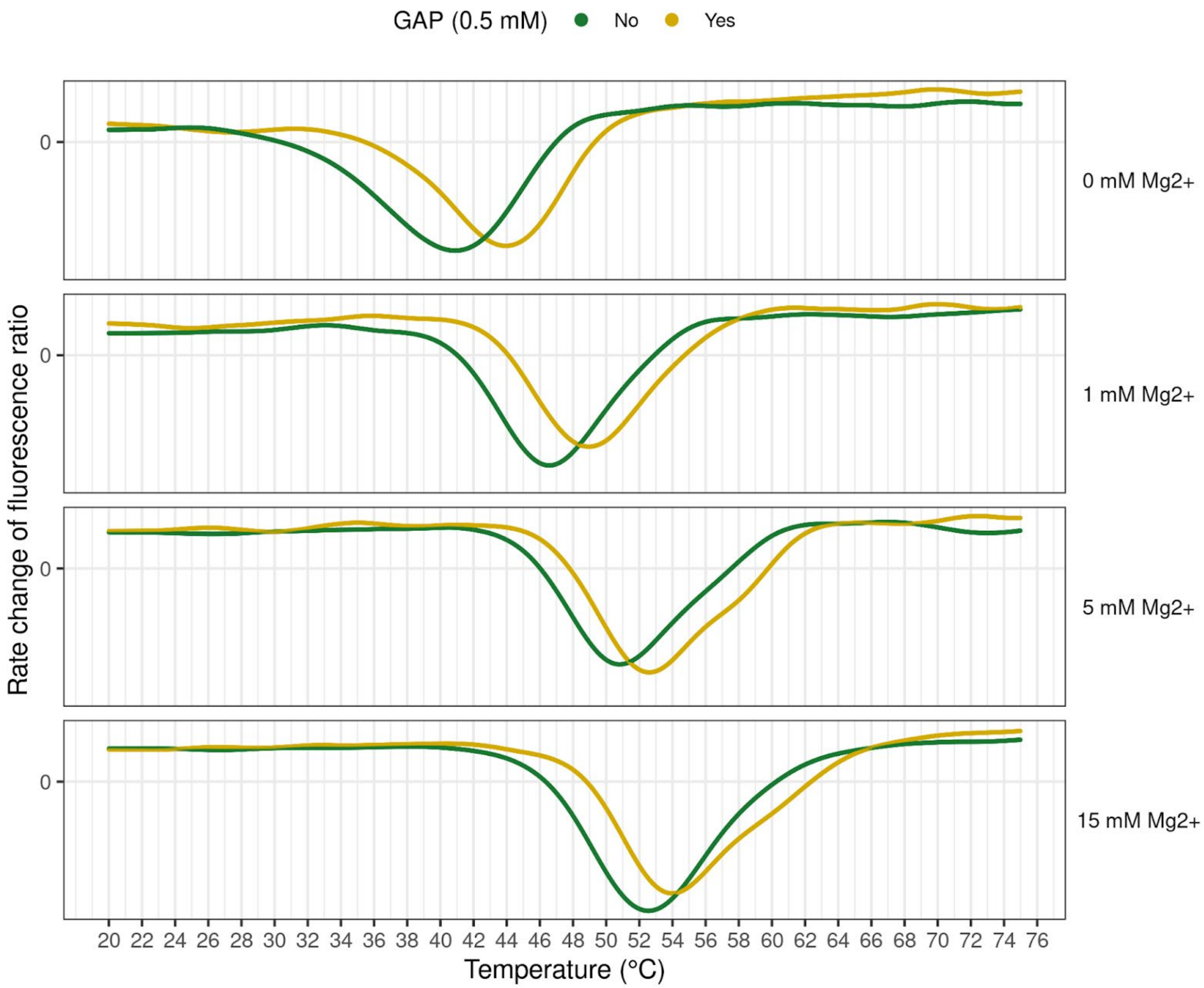

Fig. S11. Glyceraldehyde-3-phosphate (GAP) effect on thermal stability of *Synechocystis* F/SBPase at different Mg^{2+} concentrations. Curves indicate denaturation of F/SBPase over a temperature gradient of 1 °C/min. The Y-axis shows the change in the ratio of protein autofluorescence (350 nm/330 nm), and the temperature at minimum values indicate the melting temperature (T_m) at which half of the enzyme population is denatured.

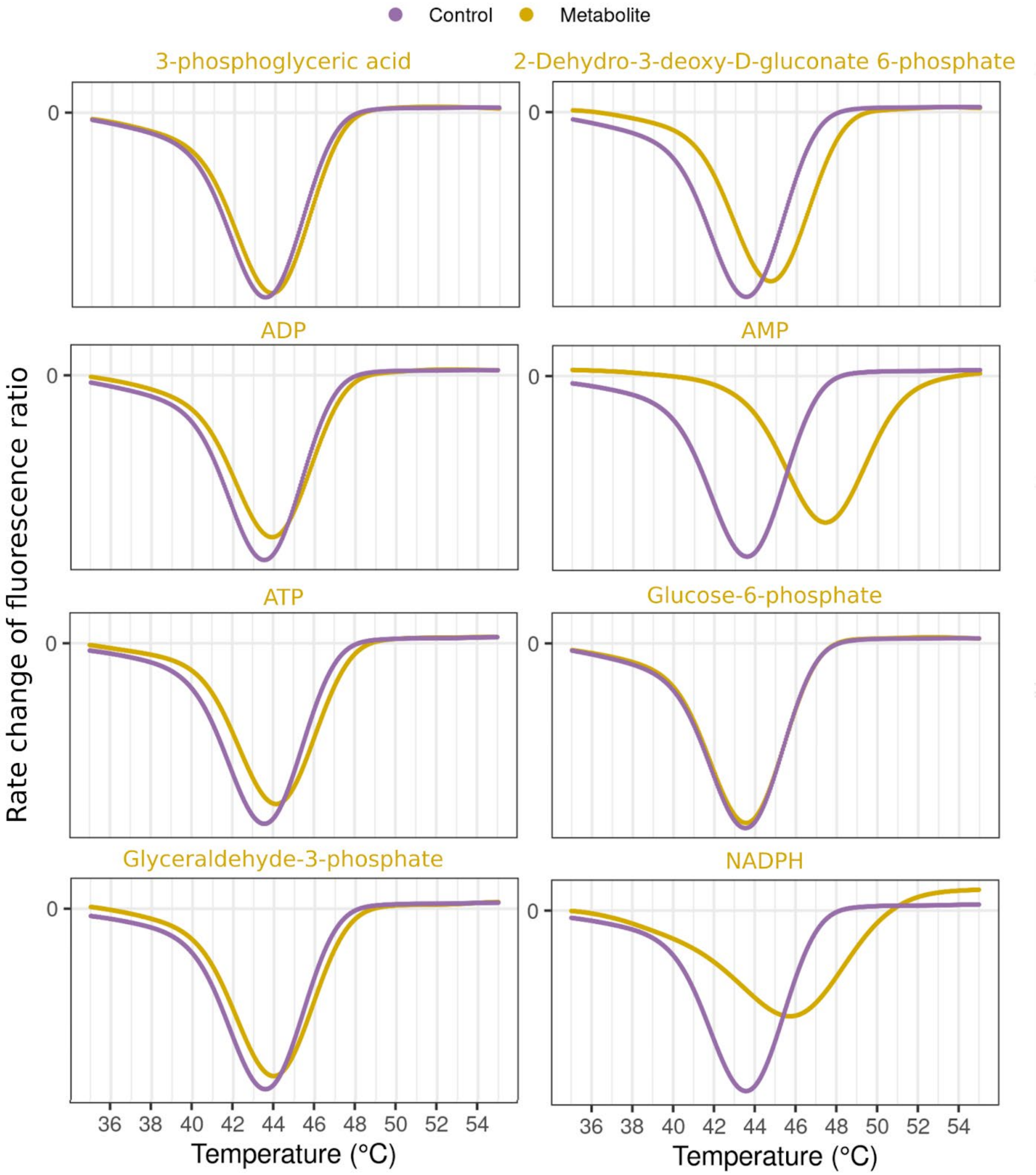

Fig. S12. Thermal shift assays of *Cupriavidus* F/SBPase in the presence of various metabolites. All metabolites were tested at a concentration of 1 mM. Curves indicate denaturation of F/SBPase over a temperature gradient of 1 °C/min. Y-axis shows the change in the ratio of protein autofluorescence (350 nm/330 nm), and minimum values indicate the melting temperature (T_m) at which half of the enzymes are denatured.

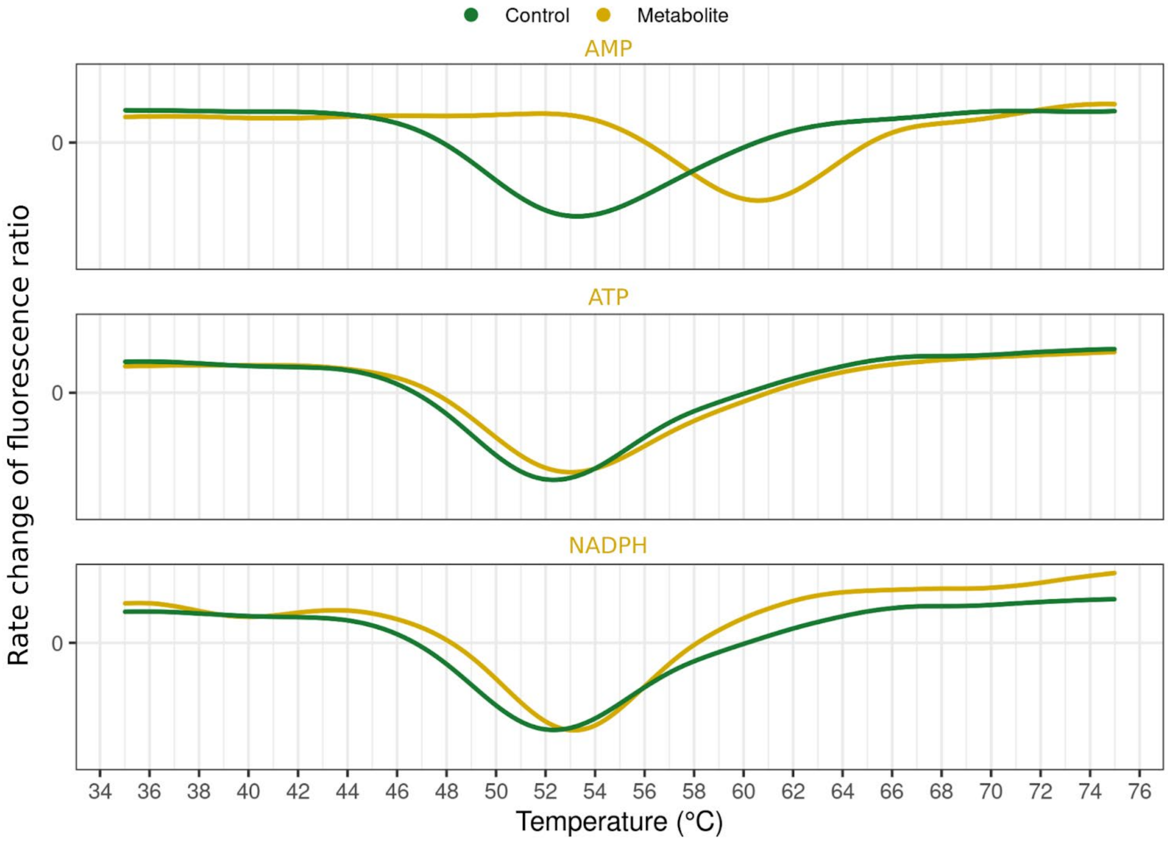

Fig. S13. Thermal shift assays of *Synechocystis* F/SBPase in the presence of various metabolites. All metabolites were tested at a concentration of 1 mM. Curves indicate denaturation of F/SBPase over a temperature gradient of 1 °C/min. Y-axis shows the change in the ratio of protein autofluorescence (350 nm/330 nm), and minimum values indicate the melting temperature (T_m) at which half of the enzymes are denatured.

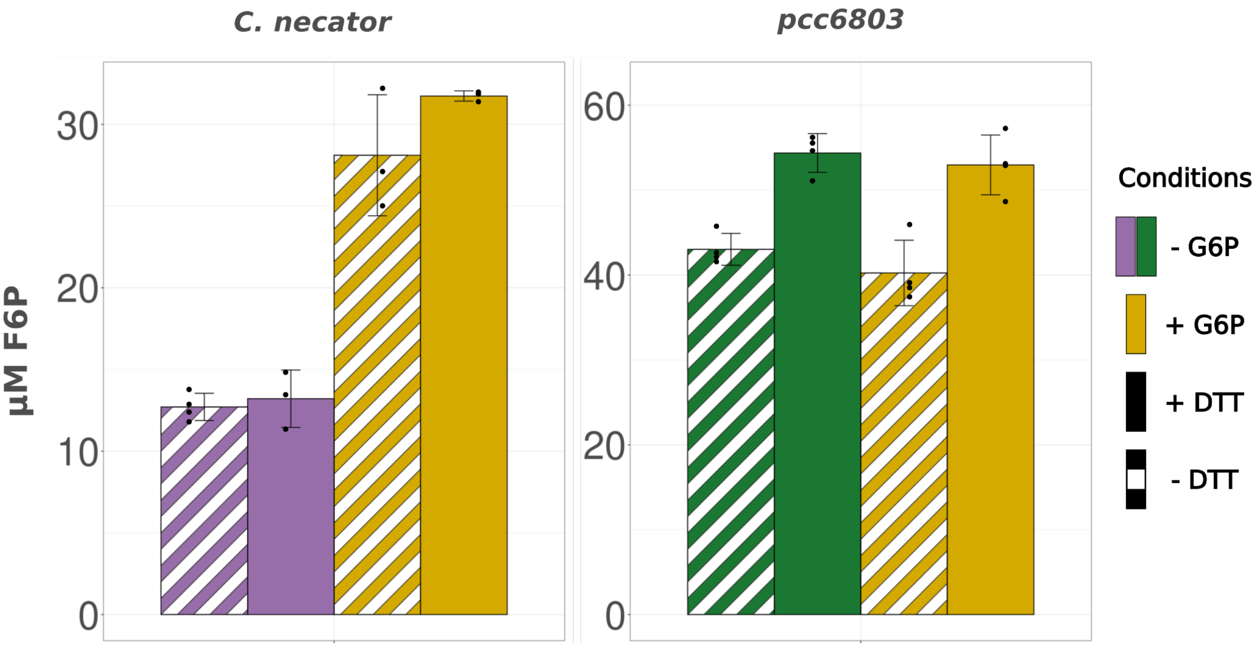

Fig. S14. End-point *in vitro* assay of F/SBPase in presence of 1 mM G6P. FBP concentration was 150 µM and enzyme concentration was 0.15 ng/µL for cn-F/SBPase and 0.45 ng/µL for syn-F/SBPase. The method of detection was a Malachite Green assay of released P_i after 20 minutes of reaction. The error bars represent the standard deviation of the data.

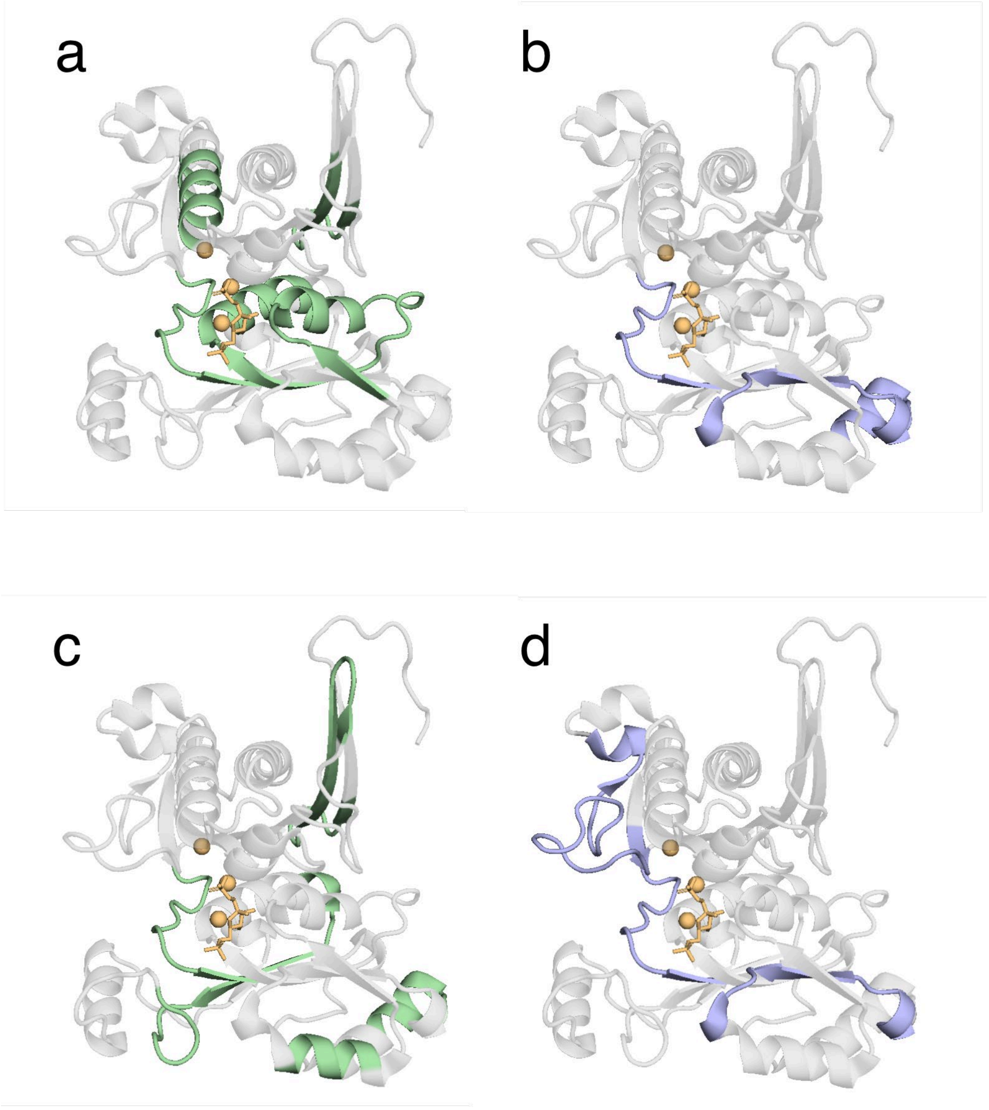

Fig. S15. Comparison of affected peptides from LiP of syn-F/SBPase for purified protein and from proteome extracts. A) Highlight of peptides (colored green) significantly affected in a LiP experiment with GAP treatment (0.5 mM), purified syn-F/SBPase. B) Highlight of peptides (purple) affected in Lip experiment with GAP treatment (0.5 mM), syn-F/SBPase in *Synechocystis* proteome extracts. C) Highlight of peptides (green) affected in Lip experiment with NADPH treatment (3 mM), purified syn-F/SBPase. D) Highlight of peptides (purple) affected in Lip experiment with NADPH treatment (3 mM), syn-F/SBPase in *Synechocystis* proteome extracts. Syn-F/SBPase structure is the monomer from PDB 3RPL. The FBP substrate is in sticks and Mg^{2+} are in yellow spheres.

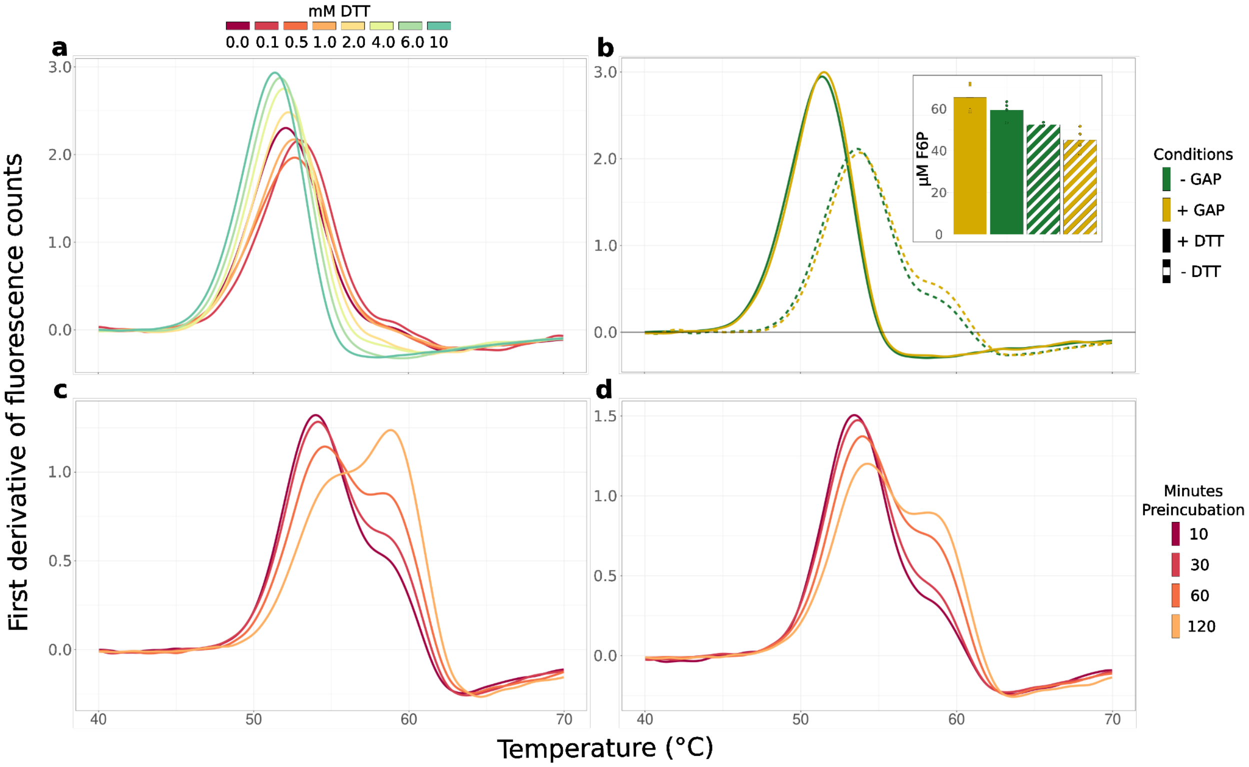

Fig. S16. Light scattering assays of syn-F/SBPase under various conditions. a Light scattering data of the enzyme at different concentrations of DTT ranging from 0 to 10 mM. **b** Light scattering data for enzyme ± 10 mM DTT and ± 0.5 mM GAP. Also shown is the concentration of product measured by malachite green assay after 20 minutes of reaction as described under Methods **c** Light scattering data of enzyme after different preincubation times at 30 degrees in the presence of 0.5 mM GAP. **d** Light scattering data of enzyme after different preincubation times at 30 degrees in the absence of GAP.

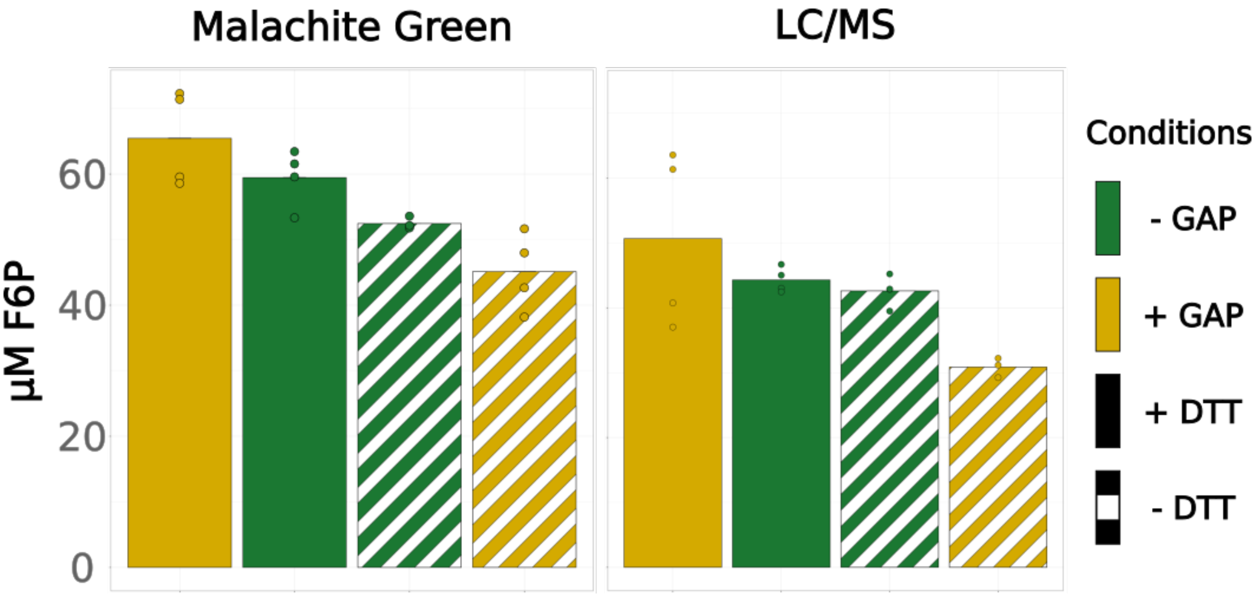

Fig. S17. Measured concentrations of product after 20 minutes of *in vitro* reaction as detected by malachite green assay and LC/MS. A substrate concentration of 80 µM was used and the enzyme concentration was 0.23 ng/µL. GAP inhibits activity in absence of DTT ($p = 0.0005$, LC/MS) and increases activity in the presence of GAP ($p = 0.42$, LC/MS).

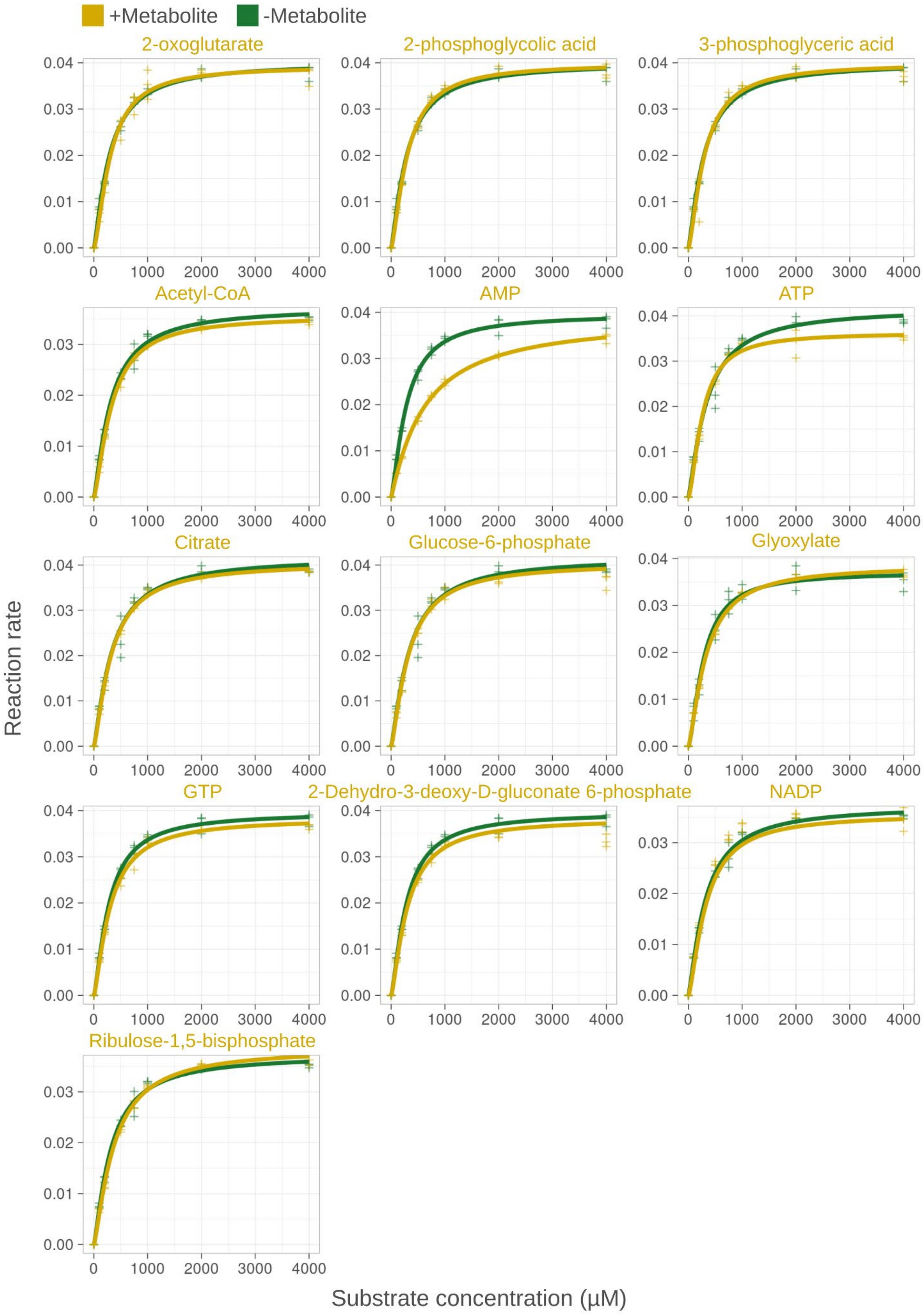
+Metabolite
-Metabolite
2-oxoglutarate
2-phosphoglycolic acid
3-phosphoglyceric acid
Acetyl-CoA
AMP
ATP
Citrate
Glucose-6-phosphate
Glyoxylate
GTP
2-Dehydro-3-deoxy-D-gluconate 6-phosphate
NADP
Ribulose-1,5-bisphosphate
Reaction rate
Substrate concentration (µM)

Fig. S18. Effect of different metabolites (1 mM) on the kinetics of *Synechocystis* transketolase. The conversion of D-ribose-5-phosphate (substrate) and L-erythrulose to sedoheptulose-7-phosphate and glycolaldehyde was measured through the consumption of NADH by alcohol dehydrogenase when reducing glycolaldehyde to ethylene glycol. Each kinetic profile was characterized by measuring reaction rates for eight different substrates. Each kinetic profile was characterized by measuring reaction rates at eight different substrate concentrations in triplicates. Separate control reactions were run in parallel for each metabolite test. Kinetic data shown in Table S4.

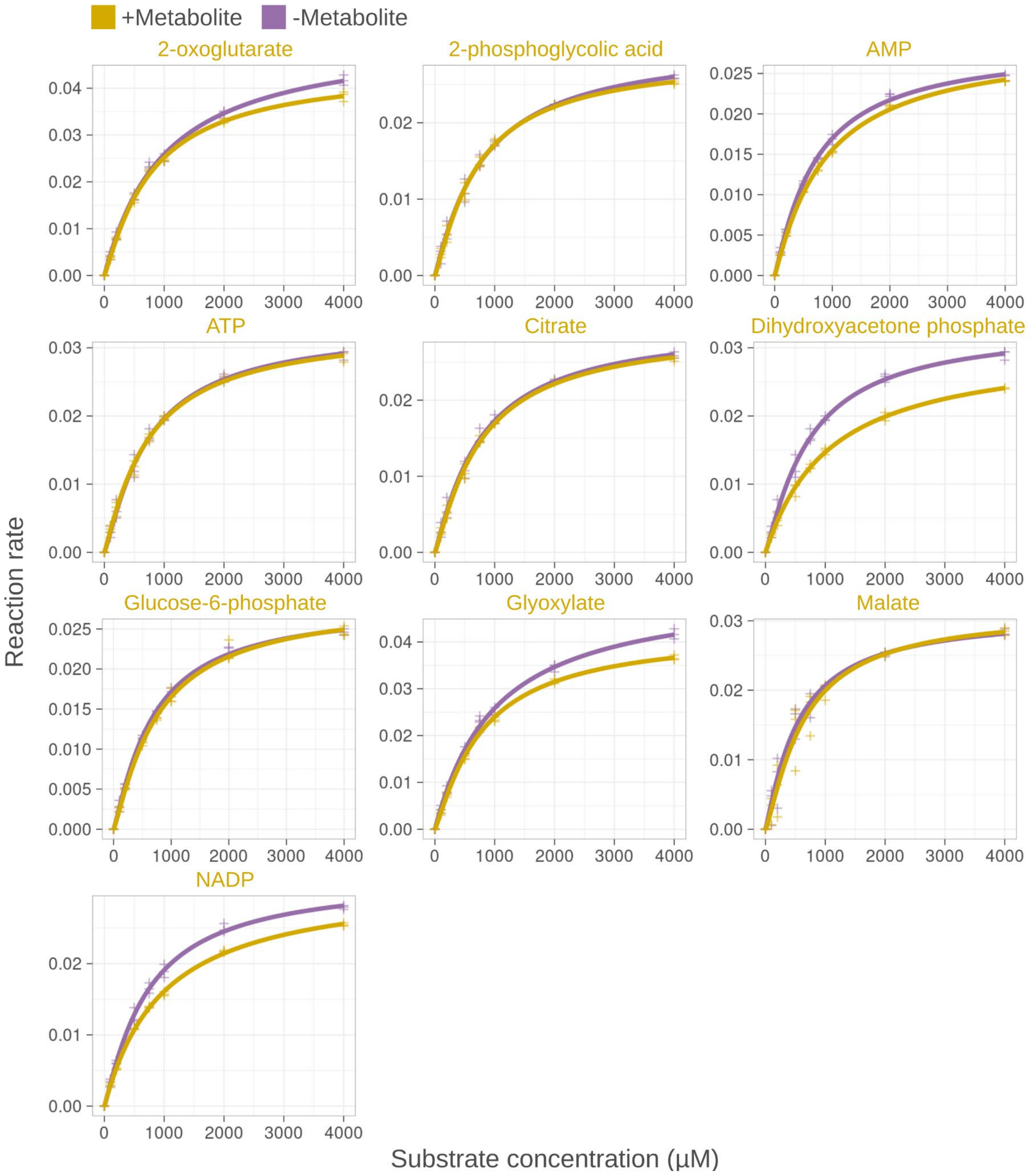

Fig. S19. Effect of different metabolites (1 mM) on the kinetics of *Cupriavidus* transketolase. The conversion of D-ribose-5-phosphate (substrate) and L-erythrulose to sedoheptulose-7-phosphate and glycolaldehyde was measured through the consumption of NADH by alcohol dehydrogenase when reducing glycolaldehyde to ethylene glycol in a coupled spectrophotometric enzyme assay.g Initial rates (uM/sec) were measured at eight different

substrate concentrations in triplicates. Lines represent data fit to the enzymatic Hill equation. Kinetic data shown in Table S4.

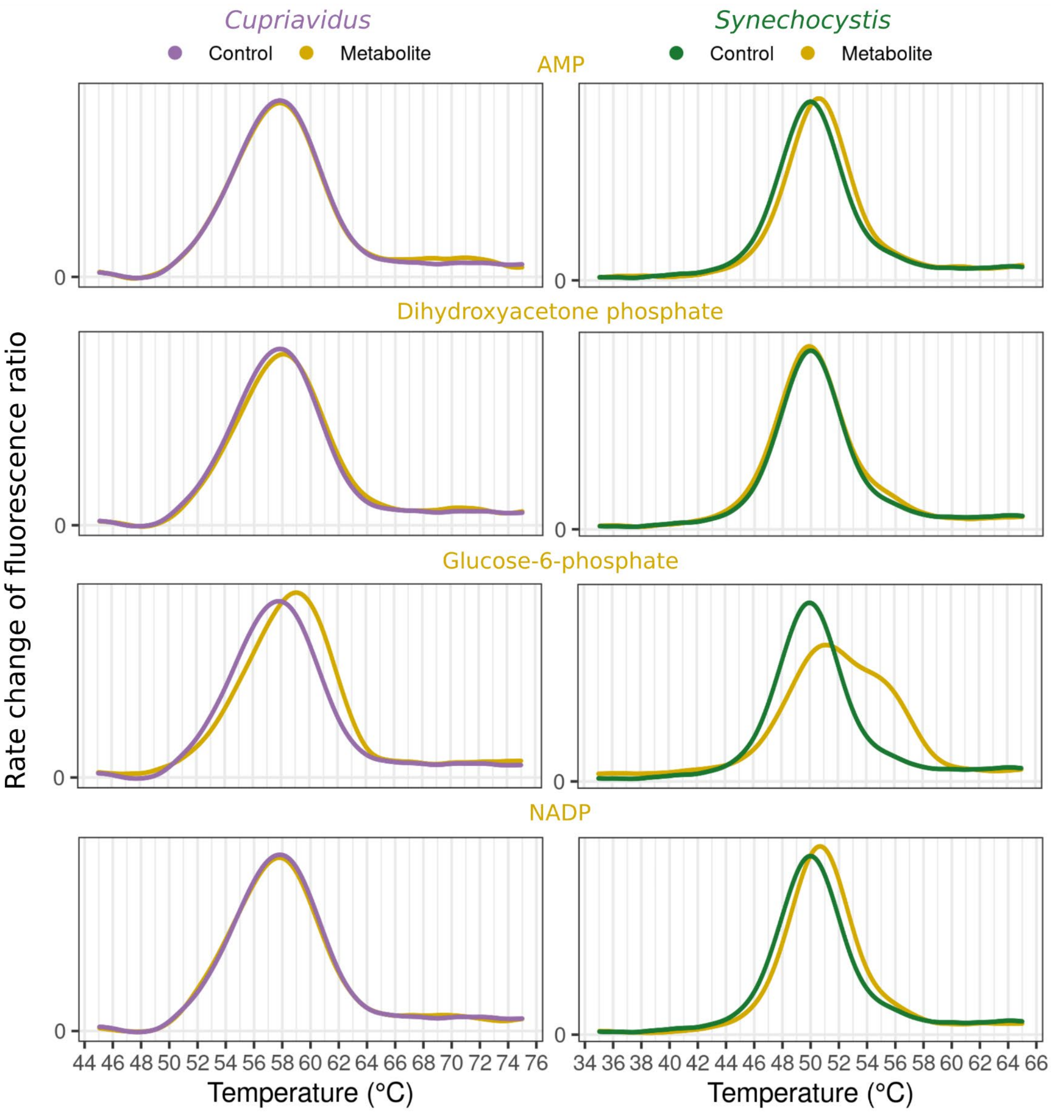

Fig. S20. Thermal shift assays of *Synechocystis* and *Cupriavidus* transketolase in the presence of various metabolites. All metabolites were tested at a concentration of 1 mM. Curves indicate denaturation of transketolase over a temperature gradient of 1 °C/min. Y-axis shows the change in the ratio of protein autofluorescence (350 nm/330 nm), and maximum values indicate the melting temperature (T_m) at which half of the enzymes are denatured.

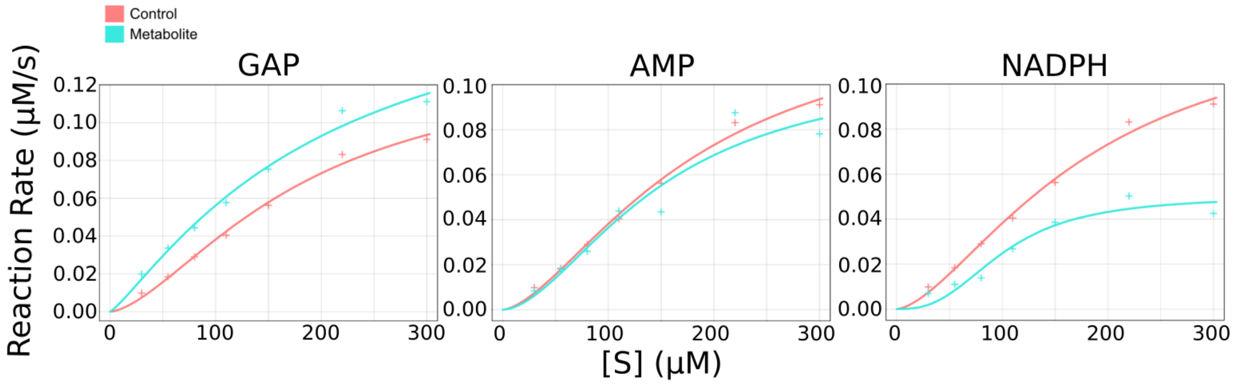

Fig. S21. Kinetic analysis of the *Synechocystis* F/SBPase R194H mutant. The enzyme is AMP insensitive, but retains sensitivity to GAP and NADPH, consistent with binding sites of GAP and NADPH as detected by LiP being distinct from AMP binding site.

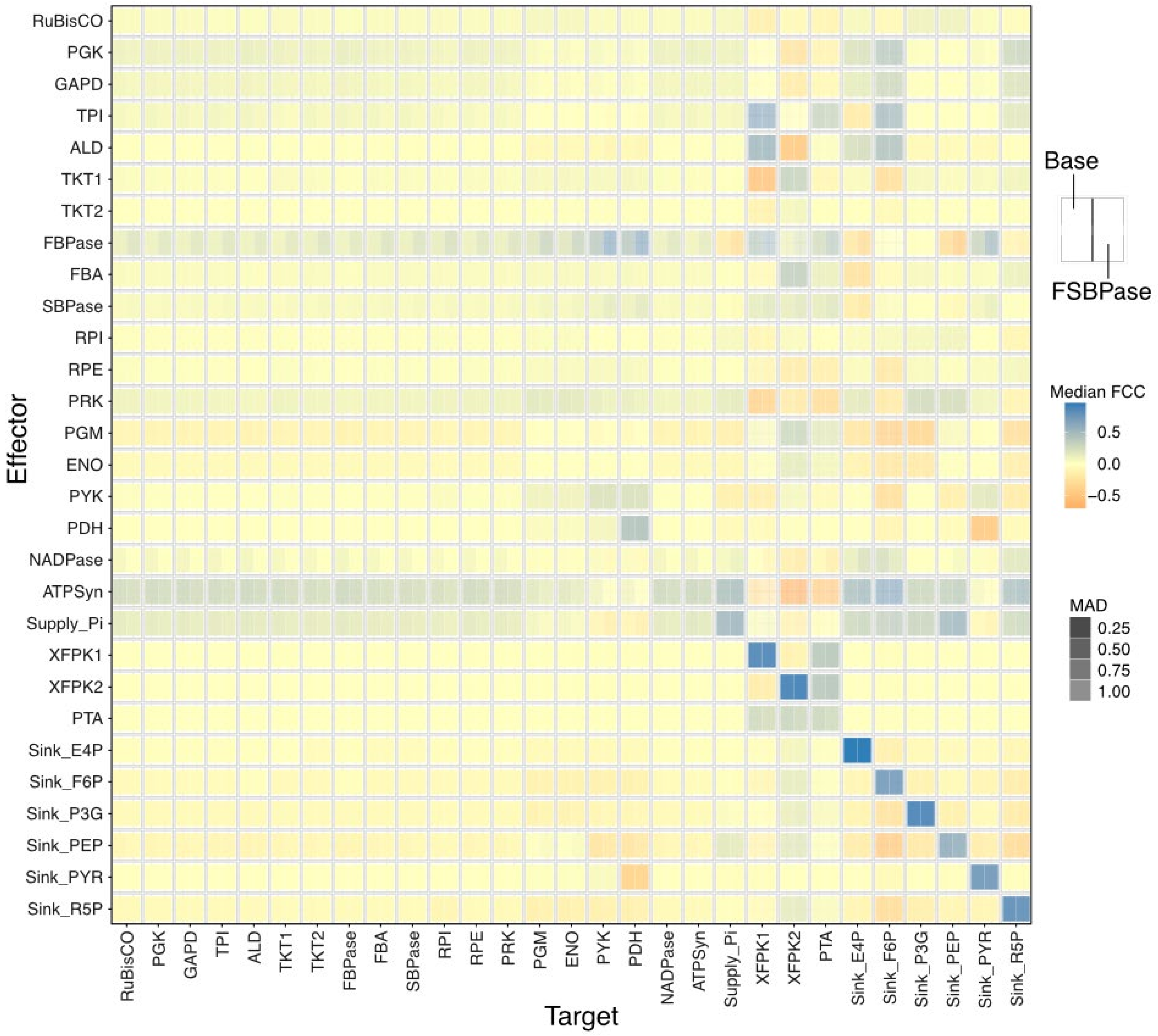

Fig. S22. Flux control coefficients for all reactions in the model. Median FCCs and MAD values were calculated over all stable parameter sets for both variants as described in the methods section.

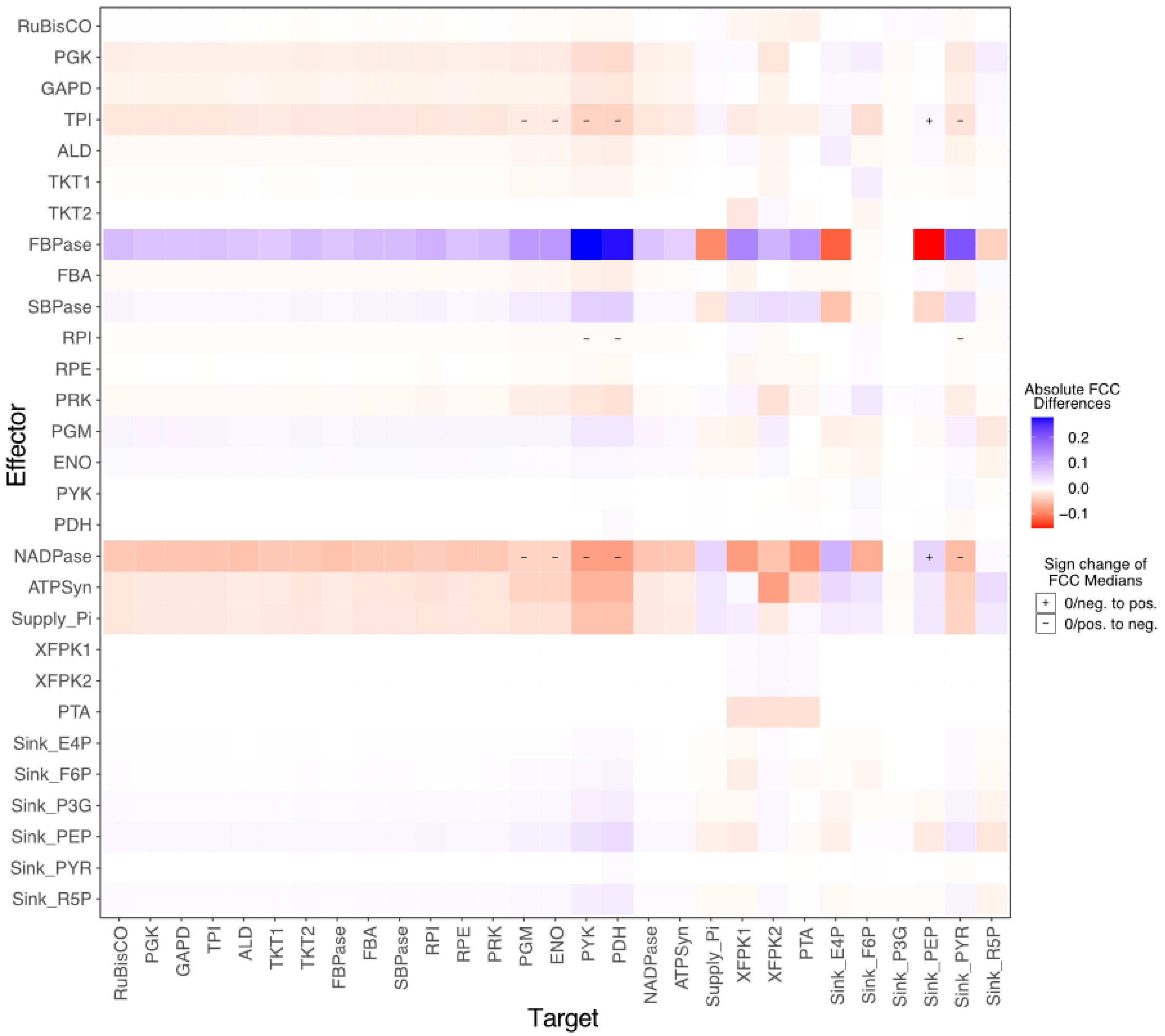

Fig. S23. Difference between median FCCs between model variants. Median FCC of F/SBPase model subtracted by median FCC of base model for each Target/Effector pair. Plus signs indicate cases where the median FCC changed to a positive influence upon added F/SBPase regulation, whereas minus signs indicate cases where the FCC changed to a negative influence.

Table S1. Chosen concentrations (mM) for every used metabolite and boundary values found in literature.
The metabolite concentrations chosen for the LiP-SMap experiments in mM, the highest and lowest concentration found in literature, in mM (**Table S2**), and the highest and lowest concentrations allowed in the thermodynamically constrained model of Asplund-Samuelsson et al. 2018 [1]. In addition to the values shown below, several other reports have shown that metabolite concentrations can vary strongly across different conditions [2,3]. As such, the high tested concentration was generally set higher than reported cellular concentration to capture metabolite accumulation that may occur during nutrient stress or metabolic perturbations and act as a regulatory signal.

Metabolite		Concentration ranges in literature				Chosen concentrations	
**Name**	**KEGG**	**Literature range lower limit**	**Literature range upper limit**	**Modeling range lower limit**	**Modeling range upper limit**	**Low**	**High**
2-phosphoglycolate	C00988	0.17	0.17	0.0001	100.0000	0.2	4
2-oxoglutarate	C00026	0.19	3.4	0.0031	2.1200	1	10
3-phosphoglycerate	C00597	1.54	19.02	-	-	2	20
6-phosphogluconate	C00345	0.07	9.58	0.0070	16.3800	1	10
Acetyl-CoA	C00024	0.01	1.54	0.0001	0.9640	1	10
ADP	C00008	0.58	5.26	0.0428	4.1867	1	10
AMP	C00020	0.34	13.4	0.0470	11.2000	1	10
ATP	C00002	0.2	3.06	0.0300	43.4300	2	32
cAMP	C00575	0.34	0.34	-	-	0.5	5
Citrate	C00158	0.17	5.45	0.0240	2.4800	2	20
Fructose-1,6-bisphosphate	C00354	0.17	4.97	0.0163	7.0694	1	10
Glucose-6-phosphate	C00668	0.17	3.4	-	-	1	10
Glyceraldehyde-3-phosphate	C00118	0.17	0.65	0.0001	100.0000	0.5	5

Glycolate	C00160	0.09	0.17	-	-	1	10
Glyoxylate	C00048	0.17	0.17	0.0001	100.000	1	10
GTP	C00044	0.14	4.87	0.1595	1.0358	1	10
KDPG	C04442	-	-	-	-	0.5	5
Malate	C00149	0.17	6.79	0.0142	2.0602	1	10
NADP	C00006	0.17	2.14	0.0055	1.3200	0.5	5
NADPH	C00005	0.14	0.24	0.0001	49.4100	0.5	5
Phosphoenolpyruvate	C00074	0.51	6.79	0.1700	2.9900	0.5	10
Phenylalanine	C00079	0.12	0.25	0.0151	0.0955	0.5	5
Ribulose-5-phosphate	C00199	0.03	5.09	0.0077	3.8900	1	10
Ribulose-1,5-bisphosphate	C01182	0.05	17.15	0.0001	11.2311	1	10
Sucrose	C00089	-	-	-	-	1	10

Table S2. All metabolite concentrations found across 7 metabolomics studies in mM.
Absolute metabolite concentrations found in literature. All values obtained from articles studying cyanobacteria were converted from µmol per gram cell dry weight to millimolar. This was done by calculating the amount of cell volume per gram dry weight from the values reported by Zavřel et al. for a growth rate of 0.05 h^{-1} [4]. The cell volume was calculated from the cell diameter and multiplied by the cell count per liter culture to obtain the total cell volume per liter culture. This was then divided by the dry weight per liter cell culture, giving the amount of cell volume per dry weight.

**Metabolite**		**E.coli**	**PCC 6803**					
**Name**	**KEGG**	**Bennet (2009)**	**Nishiguchi (2019)**	**Yoshikawa (2013)**	**Takahashi (2008)**	**Shastri (2007)**	**Hasunuma (2013)**	**Dempo (2014)**
2-phosphoglycolate	C00988	-	-	-	-	0.17	-	-
2-oxoglutarate	C00026	0.44	-	0.71	0.19	3.40	-	-
3-phosphoglycerate	C00597	1.54	12.24	2.26	2.38	10.19	2.21	19.02
6-phosphogluconate	C00345	1.64	0.07	0.15	0.08	0.17	-	9.58
Acetyl-CoA	C00024	0.73	1.54	0.27	-	0.17	0.01	0.70
ADP	C00008	0.56	0.58	1.12	0.85	-	1.87	5.26
AMP	C00020	0.28	-	0.76	0.34	-	1.51	13.40
ATP	C00002	9.63	1.34	13.12	1.02	-	0.20	3.06
cAMP	C00575	0.08	-	-	-	-	0.34	-
Citrate	C00158	0.85	5.45	1.70	0.85	0.17	-	0.42
Fructose-1,6-bisphosphate	C00354	15.20	0.51	4.97	0.17	0.17	-	0.42
Glucose-6-phosphate	C00668	-	1.70	2.46	0.17	3.40	0.49	3.24
Glyceraldehyde-3-phosphate	C00118	-	-	-	0.17	0.17	-	0.65

Glycolate	C00160	-	-	-	-	0.17	-	0.09
Glyoxylate	C00048	-	-	-	-	0.17	-	-
GTP	C00044	4.87	-	0.90	-	-	-	0.14
KDPG	C04442	-	-	0.00	-	-	-	-
Malate	C00149	-	0.49	0.48	0.17	6.79	0.19	0.20
NADP	C00006	0.00	2.14	1.17	0.17	-	-	0.97
NADPH	C00005	0.12	0.14	-	0.24	-	-	-
Phosphoenolpyruvate	C00074	0.18	6.79	1.49	0.51	6.79	1.09	2.90
Phenylalanine	C00079	0.04	-	0.12	-	-	0.13	0.25
Ribulose-5-phosphate	C00199	-	0.02	0.37	0.10	5.09	0.05	0.03
Ribulose-1,5-bisphosphate	C01182	-	0.85	5.91	0.05	17.15	-	0.48
Sucrose	C00089	-	-	-	-	-	-	-

References: [5–11]

Table S3. Changes in F/SBPase kinetic parameters in the presence of metabolites. See separate file: TableS3_summary_stats_FSBPase_kinetics.xlsx. Table shows mean and standard deviation of kinetic parameters for syn-F/SBPase and cn-F/SBPase, with and without added metabolite (2-3 replicate assays). P-values were calculated by comparing kinetic parameters with versus without added metabolite, using Student's t-tests. The columns named "*Max rate change"* and "*2nd highest rate change"* show the maximum and 2nd highest change in catalytic rate when metabolite is added, across all tested substrate concentrations.

**Synechocystis, full kinetics**								
			**Kinetic parameters (mean)**			**Kinetic parameters (Std. dev.)**		
**Experiment**	**Detection method**	**Metabolite added**	**Km**	**kcat**	**Hill**	**Km**	**kcat**	**Hill**
AcCoA (2 mM)	Malachite Green	No	45,101	8,399	1,094	4,626	0,234	0,125
AcCoA (2 mM)	Malachite Green	Yes	28,441	7,039	1,282	0,545	0,713	0,011
AMP (0.25 mM)	Malachite Green	No	67,292	5,827	1,303	1,263	0,704	0,145
AMP (0.25 mM)	Malachite Green	Yes	NA	NA	NA	NA	NA	NA
Citrate (5 mM)	Malachite Green	No	77,418	8,357	1,238	14,567	0,240	0,147
Citrate (5 mM)	Malachite Green	Yes	112,708	7,419	1,164	14,009	0,520	0,119
GAP, + DTT (0.5 mM)	Malachite Green	No	66,176	6,479	1,197	8,889	0,366	0,159
GAP, + DTT (0.5 mM)	Malachite Green	Yes	31,988	6,216	1,421	3,411	0,356	0,180
NADPH (3 mM)	Malachite Green	No	66,672	6,865	1,175	17,643	0,975	0,120
NADPH (3 mM)	Malachite Green	Yes	40,594	4,603	1,434	7,172	0,631	0,081
	**Kinetic parameters without metabolite (mean)**			**Comparsion +metabolite vs -metabolite**				
**Experiment**	**Km**	**kcat**	**Hill**	**Km_pval**	**kcat_pval**	**Hill_pval**	**Max rate change (%)**	**2nd highest rate change (%)**
AcCoA	45,101	8,399	1,094	0,121	0,067	0,278	10,987	−9,750
AMP	67,292	5,827	1,303	NA	NA	NA	−100,000	−100,000
Citrate	77,418	8,357	1,238	0,039	0,071	0,532	−39,952	−30,640
GAP	66,176	6,479	1,197	0,013	0,423	0,182	60,601	48,068
NADPH	66,672	6,865	1,175	0,110	0,035	0,044	−24,209	−23,485
Mean	64,532	7,185	1,201	-	-	-	-	-
Std. dev.	11,816	1,150	0,077	-	-	-	-	-
**Cupriavidus, full kinetics**								
			**Kinetic parameters (mean)**			**Kinetic parameters (Std. dev.)**		

Experiment	Detection method	Metabolite added	Km	kcat	Hill	Km	kcat	Hill
AMP (0.25 mM)	Malachite Green	No	49,019	16,185	1,590	10,186	2,932	0,853
AMP (0.25 mM)	Malachite Green	Yes	41,412	11,561	1,587	3,828	0,858	0,525
GAP (0.5 mM)	Malachite Green	No	33,550	10,542	1,567	4,074	0,768	0,123
GAP (0.5 mM)	Malachite Green	Yes	21,019	11,488	2,024	5,564	1,368	0,366
NADPH (3 mM)	Malachite Green	No	24,957	18,769	1,782	4,528	0,939	0,444
NADPH (3 mM)	Malachite Green	Yes	12,701	12,156	1,312	2,637	0,346	0,360

	Kinetic parameters without metabolite (mean)			Comparsion +metabolite vs -metabolite				
Experiment	Km	kcat	Hill	Km_pval	kcat_pval	Hill_pval	Max rate change (%)	2nd highest rate change (%)
AMP	49,019	16,185	1,590	0,473	0,249	0,997	−26,638	−25,223
GAP	33,550	10,542	1,567	0,039	0,370	0,154	54,101	39,530
NADPH	24,957	18,769	1,782	0,024	0,003	0,230	−36,321	−35,746
Mean	35,842	15,165	1,646					
Std. dev.	12,194	4,207	0,118					

Synechocystis, single substrate concentration

Experiment	Detection method	Metabolite added	uM F6P after 20 min	sd	pval (+metabolite vs - metabolite)
GAP, - DTT (0.5 mM)	Malachite Green	No	52,455	0,754	
GAP, - DTT (0.5 mM)	Malachite Green	Yes	45,118	5,919	0,088
GAP, - DTT (0.5 mM)	LC/MS	No	42,622	2,357	
GAP, - DTT (0.5 mM)	LC/MS	Yes	30,880	1,511	0,001
G6P, - DTT (2mM)	Malachite Green	No	17,508	1,074	
G6P, - DTT (2mM)	Malachite Green	Yes	17,272	1,268	0,786
G6P, + DTT (2mM)	Malachite Green	No	26,788	1,463	
G6P, + DTT (2mM)	Malachite Green	Yes	25,455	1,004	0,190

Cupriavidus, single substrate concentration

Experiment	Detection method	Metabolite added	uM F6P after 20 min	sd	pval (+metabolite vs - metabolite)

G6P, - DTT (2mM)	Malachite Green	No	8,089	3,015	
G6P, - DTT (2mM)	Malachite Green	Yes	14,726	1,694	0,014
G6P, + DTT (2mM)	Malachite Green	No	8,168	1,612	
G6P, + DTT (2mM)	Malachite Green	Yes	22,744	2,343	0,002

Table S4. Changes in transketolase kinetic parameters in the presence of various metabolites at 1 mM. Table shows mean and standard deviation of kinetic parameters for syn-TKL and cn-TKL with added metabolite as compared to a control without added metabolite (4 replicate assays). P-values were calculated by comparing kinetic parameters with versus without added metabolite, using Student's t-tests.

**Added Metabolite (1mM)**	**Mean V_{max} $_{R5P}$ uM/ng*sec**	**V_{max} s.d.**	**V_{max} p-value**	**Mean K_M $_{R5P}$ uM**	**K_M s.d.**	**K_M p-value**
Synechocystis tktA						
2OG	0.041	0.002	0.61	290	4	0.88
2PG	0.041	0.001	0.72	310	15	0.39
3PGA	0.040	0.001	0.75	310	14	0.40
**AcCoA**	**0.035**	**0.001**	**0.02**	320	13	0.56
**AMP**	0.039	0.002	0.53	**620**	**80**	**0.02**
**ATP**	**0.037**	**0.001**	**0.01**	260	12	0.1
Cit	0.040	0.001	0.11	320	6	0.60
**G6P**	**0.037**	**0.001**	**0.01**	20	28	0.20
Glyx	0.038	0.001	0.21	330	18	0.16
GTP	0.039	0.001	0.60	320	20	0.13
**KDPG**	**0.037**	**0.001**	**0.05**	290	10	0.83
NADP	0.036	0.002	0.30	250	20	0.06
RuBP	0.039	0.001	0.17	370	27	0.08
Cupriavidus cbbTP						
2OG	0.029	0.001	0.82	810	69	0.52
**2PG**	0.028	0.001	0.26	**700**	**14**	**0.03**
AMP	0.025	0.0001	0.47	1000	51	0.06
ATP	0.024	0.001	0.11	770	57	0.22
Cit	0.029	0.001	0.82	790	96	0.74
**DHAP**	0.031	0.002	0.11	**1100**	**116**	**0.02**
G6P	0.028	0.002	0.43	790	100	0.14
Glyx	0.031	0.03	0.31	770	153	0.85
Mal	0.029	0.0001	0.76	780	14	0.22
**NADP**	0.031	0.0005	0.38	**970**	**28**	**0.03**

Table S5. Transition list for mass spectrometry.

Compound	Retention Time (min)	RT window (min)	Precursor (m/z)	Product (m/z)	Collision Energy (V)	Min Dwell Time (ms)
F6P	4.5	9	259.022	138.979	20	38.505
F6P	4.5	9	259.022	96.969	35	38.505
F6P	4.5	9	259.022	78.959	40	38.505

Supplementary References

1. Asplund-Samuelsson, J., Janasch, M. & Hudson, E. P. Thermodynamic analysis of computed pathways integrated into the metabolic networks of E. coli and Synechocystis reveals contrasting expansion potential. *Metab. Eng.* **45**, 223–236 (2018).
2. Lempp, M. *et al.* Systematic identification of metabolites controlling gene expression in E. coli. *Nat. Commun.* **10**, 4463 (2019).
3. Marcus, Y., Harel, E. & Kaplan, A. Adaptation of the Cyanobacterium Anabaena variabilis to Low CO(2) Concentration in Their Environment. *Plant Physiol.* **71**, 208–210 (1983).
4. Zavřel, T. *et al.* Quantitative insights into the cyanobacterial cell economy. *Elife* **8**, (2019).
5. Bennett, B. D. *et al.* Absolute metabolite concentrations and implied enzyme active site occupancy in Escherichia coli. *Nat. Chem. Biol.* **5**, 593–599 (2009).
6. Nishiguchi, H. *et al.* Transomics data-driven, ensemble kinetic modeling for system-level understanding and engineering of the cyanobacteria central metabolism. *Metab. Eng.* **52**, 273–283 (2019).
7. Yoshikawa, K. *et al.* Integrated transcriptomic and metabolomic analysis of the central metabolism of Synechocystis sp. PCC 6803 under different trophic conditions. *Biotechnol. J.* **8**, 571–580 (2013).
8. Shastri, A. A. & Morgan, J. A. A transient isotopic labeling methodology for 13C metabolic flux analysis of photoautotrophic microorganisms. *Phytochemistry* **68**, 2302–2312 (2007).
9. Hasunuma, T. *et al.* Dynamic metabolic profiling of cyanobacterial glycogen biosynthesis under conditions of nitrate depletion. *J. Exp. Bot.* **64**, 2943–2954 (2013).
10. Dempo, Y., Ohta, E., Nakayama, Y., Bamba, T. & Fukusaki, E. Molar-based targeted metabolic profiling of cyanobacterial strains with potential for biological production. *Metabolites* **4**, 499–516 (2014).

11. Takahashi, H., Uchimiya, H. & Hihara, Y. Difference in metabolite levels between photoautotrophic and photomixotrophic cultures of Synechocystis sp. PCC 6803 examined by capillary electrophoresis electrospray ionization mass spectrometry. *J. Exp. Bot.* **59**, 3009–3018 (2008).
