# Supplementary material for: Metabolite interactions in the bacterial Calvin cycle and implications for flux regulation
Source: Commun Biol. 2023 Sep 18;6:947. doi: 10.1038/s42003-023-05318-8 (PMC10507043; doi:10.1038/s42003-023-05318-8)
Supplement: Supplementary file 3 — Description of Supplementary Materials [file 42003_2023_5318_MOESM3_ESM.pdf]

Description of Additional Supplementary Files

File name: Supplementary data S1
Description: List of significantly affected proteins in Synechocystis PCC 6803 by reduction/oxidation through DTT/DTNB.

File name: Supplementary data S2
Description: Table of all detected peptides across LiPSMap experiments with fold-change and significance statistics; Synechocystis PCC 6803.

File name: Supplementary data S3
Description: Table of all detected peptides across LiPSMap experiments with fold-change and significance statistics; Synechoccocus PCC 7942.

File name: Supplementary data S4
Description: Table of all detected peptides across LiPSMap experiments with fold-change and significance statistics; Cupriavidus necator.

File name: Supplementary data S5
Description: Table of all detected peptides across LiPSMap experiments with fold-change and significance statistics; Hydrogenophaga pseudoflava.

File name: Supplementary data S6
Description: List of all proteins affected by at least one metabolite and their KEGG orthology groups (KOGs).

File name: Supplementary data S7
Description: List of all significant peptides in LiP-SMap experiment with magnesium and ATP.

File name: Supplementary data S8
Description: Phylogenetic trees of Calvin cycle enzymes labelled with detected protein-metabolite interactions.

File name: Supplementary data S9
Description: Raw data for kinetic-, melting temperature and light scattering assays.
